# Association Mapping and the Genomic Consequences of Selection in Sunflower

**DOI:** 10.1371/journal.pgen.1003378

**Published:** 2013-03-21

**Authors:** Jennifer R. Mandel, Savithri Nambeesan, John E. Bowers, Laura F. Marek, Daniel Ebert, Loren H. Rieseberg, Steven J. Knapp, John M. Burke

**Affiliations:** 1 Department of Plant Biology, Miller Plant Sciences, University of Georgia, Athens, Georgia, United States of America; 2 North Central Regional Plant Introduction Station, USDA–ARS, Ames, Iowa, United States of America; 3 Department of Botany, University of British Columbia, Vancouver, British Columbia, Canada; 4 Department of Biology, Indiana University, Bloomington, Indiana, United States of America; 5 Monsanto Company, Woodland, California, United States of America; University of Minnesota, United States of America

## Abstract

The combination of large-scale population genomic analyses and trait-based mapping approaches has the potential to provide novel insights into the evolutionary history and genome organization of crop plants. Here, we describe the detailed genotypic and phenotypic analysis of a sunflower (*Helianthus annuus* L.) association mapping population that captures nearly 90% of the allelic diversity present within the cultivated sunflower germplasm collection. We used these data to characterize overall patterns of genomic diversity and to perform association analyses on plant architecture (i.e., branching) and flowering time, successfully identifying numerous associations underlying these agronomically and evolutionarily important traits. Overall, we found variable levels of linkage disequilibrium (LD) across the genome. In general, islands of elevated LD correspond to genomic regions underlying traits that are known to have been targeted by selection during the evolution of cultivated sunflower. In many cases, these regions also showed significantly elevated levels of differentiation between the two major sunflower breeding groups, consistent with the occurrence of divergence due to strong selection. One of these regions, which harbors a major branching locus, spans a surprisingly long genetic interval (ca. 25 cM), indicating the occurrence of an extended selective sweep in an otherwise recombinogenic interval.

## Author Summary

Selection during the evolution of crop plants has resulted in dramatic phenotypic differentiation, and these same selective pressures are expected to have had a significant impact on underlying genomic diversity. Population genomic analyses, especially when coupled with trait-based mapping approaches, thus have the potential to provide unique insights into the evolution of crop plants and their genomes. In this study, we performed a genome-wide analysis of genetic variation in cultivated sunflower and used the resulting data to genetically dissect variation in plant architecture (i.e., branching) and flowering time. We found substantial variation in levels of linkage disequilibrium (LD) across the genome, with islands of elevated LD generally corresponding to genomic regions underlying traits that have been targeted by selection during the evolution of cultivated sunflower. A number of these same regions also exhibited strong population genetic differentiation across the sunflower gene pool, suggesting that they may harbor genes underlying adaptation following domestication. Our analyses also identified numerous genomic regions underlying variation in both plant architecture and flowering time, many of which fall in genomic regions that have not previously been shown to influence these traits using more traditional quantitative genetic approaches.

## Introduction

Strong selection during the evolution of crop plants has resulted in dramatic phenotypic differentiation. Undoubtedly, these same selective pressures will also have produced significant genomic consequences. Indeed, genomic regions that have been targeted by selection during crop evolution are expected to exhibit characteristic changes in their levels and/or patterns of nucleotide diversity. For example, under strong directional selection, one would expect a marked decrease in genetic variation in and near the targeted loci (e.g., *tb1* [1]; *waxy* [2]; *GIF1* [3]). However, under divergent selection, an increase in population genetic differentiation would be expected between the divergently selected lineages, coupled with localized decreases in genetic variation (e.g., [4], [5]). The extent of these effects will be jointly determined by the strength of selection and the local recombination rate [6], with stronger selection and/or reduced recombination affecting diversity across a larger chromosomal region. The use of large-scale population genomic analyses, especially when coupled with trait-based mapping approaches, thus has the potential to provide novel insights into the evolutionary history of crop plants and their genomes.

Traditional quantitative trait locus (QTL) mapping analyses have provided considerable insight into the genetic basis of the phenotypic changes that have occurred during crop evolution (e.g., [7]–[9]). This general approach is, however, somewhat limited in terms of both mapping resolution and the amount of diversity assayed. Association mapping, which involves the correlation of molecular polymorphisms with phenotypic variation in a diverse assemblage of individuals, solves both of these problems, and is thus a useful alternative to standard QTL mapping approaches [10]. Because association populations typically capture many generations of historical recombination, linkage disequilibrium (LD; the non-random association of alleles between loci) is expected to be substantially lower than in family-based mapping populations, resulting in much higher mapping resolution. Moreover, the high level of diversity in a typical association mapping population allows for the simultaneous investigation of the effects of a broad spectrum of alleles across multiple genetic backgrounds. The downside of such analyses is that structure in the focal population can produce spurious marker/trait correlations in the absence of physical linkage [11], [12]. Statistical advances have, however, made it possible to minimize the likelihood of false associations by accounting for relatedness amongst individuals (i.e., kinship) and population structure (e.g., [13]–[15]).

Ultimately, detailed insights into standing levels of nucleotide diversity, background patterns of LD, and relatedness amongst individuals within the focal population are critically important for the successful application of association mapping approaches. The use of high-density single nucleotide polymorphism (SNP) data derived from known genomic locations can facilitate the development of these insights and thus has the potential to enable the genetic dissection of important phenotypes in crop plants. Moreover, the outcomes of such analyses have potential downstream applications in marker-assisted breeding programs [16]. Here, we investigate SNP diversity, population differentiation, and the structure of LD across the 3.6 Gbp genome of sunflower (*Helianthus annuus* L.) and perform association analyses of plant architecture and flowering time in this valuable crop species.

Cultivated sunflower is a globally important oilseed crop that was domesticated from the wild, common sunflower (also *H. annuus*) approximately 4,000 years ago by Native Americans. Following its domestication, sunflower was originally used as a source of edible seeds and for a variety non-food applications (e.g., as a source of dye for textiles and for ceremonial purposes) [17]–[19]. The transformation of sunflower into an oilseed crop began in 18^th^ century in Eastern Europe where breeding efforts increasingly focused on improving oil yield in a subset of the available germplasm. Commercial production commenced in North America in the mid-20^th^ century, along with a focus on the development of sunflower as a hybrid crop. Modern sunflower is maintained in two primary breeding pools: the unbranched female (A) lines (differing only in cytoplasm from paired maintainer or B lines), and the typically recessively-branching, multi-headed male restorer (R) lines that are crossed to generate the unbranched, fertile hybrids grown by producers. In this study, we used an Illumina Infinium 10 k SNP array [20], [21] to genotype a diverse collection of publicly-available sunflower lines. We then used these data, along with phenotypic data collected from multiple locations, to analyze genome-wide patterns of genetic variation, characterize the extent of LD and population differentiation, and investigate the genetic basis of variation in plant architecture and flowering time.

## Materials and Methods

### Association population

The sunflower association mapping population utilized in this study was composed of 271 lines that have previously been shown to capture nearly 90% of the allelic diversity present within the cultivated sunflower gene pool [22]. This population is composed of accessions from the collections held by both the USDA North Central Regional Plant Introduction Station (NCRPIS) and the French National Institute for Agricultural Research (INRA) (Table S1). These accessions include numerous inbred lines and historically important open-pollinated varieties (OPVs; including high-oil Eastern European cultivars), as well as oilseed and confectionery (non-oil) accessions from elsewhere in the world. Where necessary, accessions were advanced via single-seed descent for one or two generations to minimize residual heterozygosity.

All accessions were assigned to one of ten categories based on their origin (USDA or INRA), breeding history (maintainer [B] lines = HA, typically unbranched; restorer [R] lines = RHA, typically branched), and agronomic use (oil vs. non-oil). Note that an oilseed vs. confectionery designation was not available for the INRA accessions; therefore, these were divided into INRA-derived B and R lines (denoted INRA-HA and INRA-RHA, respectively). For the USDA accessions, the following categories were defined: HA non-oil, HA oil, RHA non-oil, RHA oil, introgressed, OPVs, other non-oil, and other oil. Accessions designated ‘non-oil’ were either confectionery types, or could not be clearly defined as being oil types. The ‘introgressed’ category included accessions with a recent history of introgression from wild *Helianthus* species as indicated by the available pedigree information (e.g., [23], [24]). The OPV category included named sunflower accessions that represent open-pollinated varieties of the pre-hybrid era of sunflower breeding, including Jupiter, Manchurian, Jumbo, VIR 847, Mammoth, etc. (BS Hulke, USDA-ARS, pers. comm.) along with two Native American landraces, Hopi and Mandan. The ‘other oil’ and ‘other non-oil’ categories included accessions of each type for which a B vs. R designation could not be made.

### Field planting and design

In the spring of 2010, we planted our association mapping population in replicate at three locations: the Plant Sciences Farm in Watkinsville, GA, USA, the North Central Regional Plant Introduction Station in Ames, IA, USA, and the University of British Columbia Campus in Vancouver, BC, Canada (12 seeds/plot x 271 lines x 2 replicates x 3 locales) (Figure S1). Replicates were planted in an alpha lattice design constructed using the computer module ALPHA 6.0, available from Design Computing (http://www.designcomputing.net/).

### Genotypic characterization

Total DNA was extracted from bulked tissue collected from four individuals of each line using a CTAB extraction protocol [25]. Total DNA was quantified using Picogreen (ABI), and the quality of DNA was inspected using a Nanodrop 1000 spectrophotometer. All lines were then genotyped on an Illumina Infinium 10 k SNP array designed for cultivated sunflower. The array was designed from a large collection of sunflower ESTs and included no more than one SNP per gene [20]. Genotyping was performed according to the manufacturer's recommendations on the Illumina iScan System (Illumina Inc., San Diego, CA) at the Emory University Biomarker Service Center. Prior to hybridization of the Beadchips, DNA was diluted to 50 ng/µl and quality was assessed via UV spectrophotometry and agarose gel electrophoresis. All SNP data analyses were performed using the raw intensity data from the Illumina Beadchip and Genome Studio ver. 2011.1 (Illumina) following the methods outlined in Bowers et al. [21]. Note that only those SNPs that showed clearly interpretable clustering patterns were used in this study, thereby eliminating probes that hybridized to multiple gene copies from our dataset. Map positions were obtained from the sunflower consensus map [21].

### Genetic diversity, population structure, and relative kinship

Population-wide estimates of genetic diversity, including allele frequencies, observed heterozygosity, and unbiased gene diversity [26], were calculated using GenAlEx v. 6.4 [27].

Population structure was investigated using the Bayesian, model-based clustering algorithm implemented in the software package STRUCTURE [28]. For this analysis, we used only polymorphic SNPs with a minor allele frequency (MAF) ≥10%. Briefly, individuals were assigned to *K* population genetic clusters based on their multi-locus genotypes. Clusters were assembled so as to minimize intra-cluster Hardy-Weinberg and linkage disequilibrium and, for each individual, the proportion of membership in each cluster is estimated. We employed the admixture model without the use of prior population information (i.e., USEPOPINFO was turned off). For each analysis, we evaluated *K* = 1–12 population genetic clusters with 5 runs per *K* value and averaged the probability values across runs for each cluster. For each run, the initial burn-in period was set to 50,000 with 100,000 MCMC iterations. The most likely number of clusters was then determined using the Delta*K* method of Evanno et al. [29].

Genetic relationships amongst the cultivated sunflower accessions were also investigated graphically via principal coordinates analysis (PCoA) using GenAlEx and the same set of polymorphic SNPs (MAF ≥10%) that were used for the STRUCTURE analyses. A standard genetic distance matrix [26] was constructed based on the multi-locus genotypes. This distance matrix was then used for the PCoA, and the first two principal coordinates were graphed in two-dimensional space.

A relative kinship matrix was then estimated from this set of SNPs using the program SPAGeDi [30]. Negative values between pairs of individuals, indicating that there was less relationship than that expected between two randomly chosen individuals, were set to 0 in the resulting matrix.

### Genome-wide linkage disequilibrium and population differentiation

To investigate the extent of linkage disequilibrium (LD) across the genome, a correlation matrix of *r* ^2^ values, the squared allele frequency correlations, was constructed between all possible pairs of polymorphic loci with MAF ≥10%. Following the methods of Macdonald et al. [31], we summarized the observed *r* ^2^ values using the k-smooth function in the statistical programming language R (http://www.R-project.org/). We also visualized the extent of LD and genetic variation across the genome by averaging the *r* ^2^ and UH_e_ values, respectively, in a 5 cM sliding window across each linkage group (LG).

We calculated F_ST_ for all polymorphic SNPs between the two major heterotic groups (including 125 RHA lines and 100 HA lines) and plotted the results as a function of map position. We also performed outlier analyses on these data using the software BayeScan [32]. The program utilizes the Bayesian model from Beaumont and Balding (2004) and a reversible jump Markov chain Monte Carlo method to identify outlier loci that are putatively under selection based on F_ST_ estimates. Ten pilot runs of 5,000 iterations and an additional burn-in of 50,000 iterations were first performed. We then used 100,000 iterations to identify loci under selection based on locus-specific Bayes factors. Strong evidence for selection is indicated by a Bayes factor above 10, or log10 = 1.0 [32].

### Graphical genotypes

In order to visualize genome-wide haplotypic structure in the association population, graphical genotypes were constructed by defining haplotypic blocks of 25 or more consecutive SNPs (based on the map order from [21]) that were identical between two or more cultivars. To do this, each accession was compared to all other accessions in the dataset to determine the percentage of the SNPs contained in shared haplotypic blocks. The accession with the highest fraction of the genome shared with all other accessions in the data set was set as the “template” for genotype #1 (G1). The raw data for the template accession and all matching haplotypic blocks in other accessions were converted into G1 from the raw scores. This process was repeated for 24 additional cycles, masking the data that had previously been assigned to haplotypic blocks to produce G2, G3, …, G25. The most common genotypes were then color-coded and visually presented using spreadsheet software.

### Phenotypic analyses

The number of days to flower (DTF; calculated from the planting date) was recorded at the R-5.1 reproductive stage. The R-5 stage commences at the onset of flowering, and is divided into substages according to the percentage of disc florets that have opened; R-5.1 corresponds to the stage at which 10% of the disc florets have opened. The total number of branches per plant (hereafter referred to as “branching”) was measured in the field at the R-9 reproductive stage. This stage is regarded as physiological maturity and is characterized by the presence of yellow/brown bracts on the back of the sunflower head. Four plants per accession were scored for each replicate at each of three locations (4×271×2×3 = 6,504 plants scored).

Data were analyzed using SAS software version 9.3 (SAS Institute, Cary, NC). We calculated Pearson pairwise correlation coefficients for the branching data and the average DTF across locations using PROC CORR and corrected the resulting significance levels for multiple tests using a sequential Bonferroni correction (Holm 1979). We also analyzed our data using the GLM procedure of SAS. Because our initial analyses revealed highly significant (*P*<0.001) genotype x environment (G×E) interactions (data not shown), all subsequent analyses were performed separately by location. At each location, the entry (i.e., genotype) was treated as a fixed effect and blocks and reps were treated as random effects. For branching, the block and rep effects were not significant in the model and thus raw means were used for association testing (below). Because there were significant block and rep effects for DTF, the least-squares means (LS means) were used for the association mapping of this trait. Finally, variance components using the VARCOMP function in SAS were calculated for branching and DTF and were used to estimate broad-sense heritabilities (*H^2^*) as the total genotypic variance divided by the total phenotypic variance.

### Association mapping

Association mapping of branching and DTF were performed in the software package TASSEL v. 3.0 [33] using all SNPs with MAF ≥10%. Three different association mapping models were run for each trait including a mixed linear model (MLM) accounting for kinship (i.e., familial relatedness; K-matrix) and two MLMs using kinship and population structure as estimated via either principle component analysis (PCA) (P-matrix) or the program STRUCTURE (Q-matrix) [28], [29]. Model effects for individual SNPs were output from TASSEL for each MLM [33].

Linear model testing was performed by plotting the observed *P*-values from the association test against an expected (cumulative) probability distribution. These quantile-quantile (q-q) plots indicate the extent to which the analysis produced more significant results than expected by chance. Models that follow the expected line more closely are assumed to have produced fewer false positives. Given that non-independence of linked makers in the dataset could lead to overly conservative significance thresholds [34], we used the multiple testing correction method of Gao et al. [35] to evaluate the significance of our results. This approach accounts for correlations amongst markers while controlling the type I error rate (alpha = 0.05). Using both real and simulated data, this correction has been shown to be an efficient and accurate method of minimizing false positives in the presence of inter-marker LD.

Where possible, to enable the identification of novel genetic effects, we also compared the genetic map positions of significant associations to those of previously mapped branching and flowering time QTL. This was done by projecting QTL onto the sunflower consensus map (which, as noted above, was also used for ordering the SNPs employed in the present study) based on shared markers. Note that some previous QTL results could not be included in this comparison due to a lack of shared markers and/or differences in linkage group nomenclature. The linkage group names were standardized by Tang et al. [36], though the new naming scheme was not immediately adopted by all researchers.

## Results

### Genetic diversity, population structure, and kinship

The total number of readily scorable, bi-allelic SNPs in the focal population was 5,788. The number of SNPs with a MAF ≥10% was 5,359. Expected heterozygosity, or Nei's unbiased gene diversity, averaged 0.404±0.005 (mean ± standard error), and ranged from 0.007 to 0.5. Observed heterozygosity averaged 0.034±0.0044, and ranged from 0 to 0.38. Gene diversity and observed heterozygosity for each line classification grouping are found in Table 1.

**Table 1:** Observed and expected levels of unbiased heterozygosity in sunflower groups.

Line Class	N	Ho (SE)	UHe (SE)
INRA-HA	17	0.0060 (0.0049)	0.428 (0.039)
HA-Nonoil	48	0.027 (0.0036)	0.408 (0.023)
HA-Oil	60	0.027 (0.0020)	0.428 (0.019)
INRA- RHA	11	0.0060 (0.0080)	0.444 (0.049)
RHA-Nonoil	24	0.023 (0.0060)	0.378 (0.033)
RHA-Oil	65	0.013 (0.0020)	0.431 (0.018)
Nonoil	11	0.018 (0.013)	0.418 (0.054)
Oil	9	0.042 (0.022)	0.431 (0.055)
OPV/Landrace	11	0.13 (0.044)	0.383 (0.060)
Introgressed	15	0.18 (0.050)	0.395 (0.075)
Total	271	0.034 (0.0040)	0.404 (0.0050)

**Figure 1:**
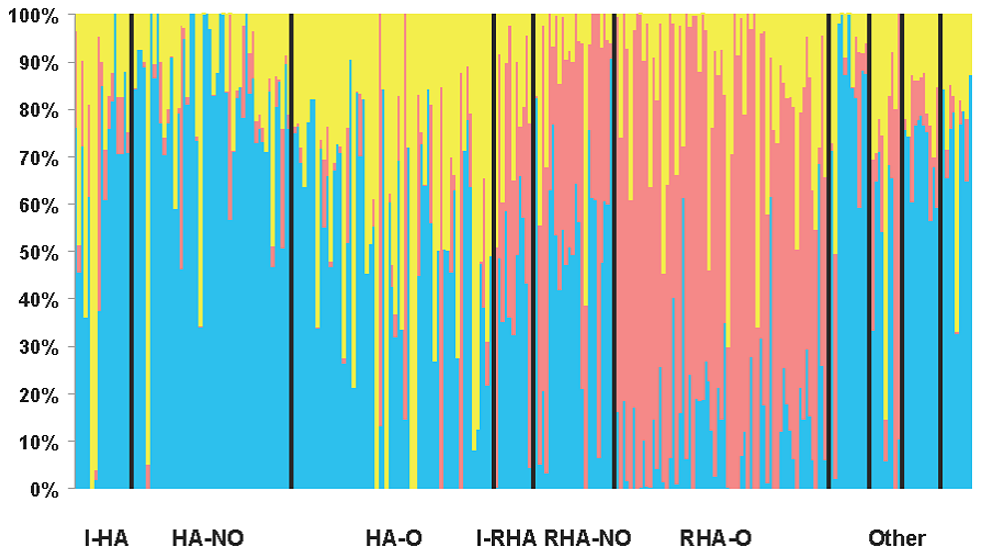
STRUCTURE plot of the sunflower association mapping panel with *K = *3 clusters based on all polymorphic SNP markers. The plot is sorted according to line classification. I-HA = INRA-HA, HA-NO = HA-non-oil, HA-O = HA-oil, I-RHA = INRA-RHA, RHA-NO = RHA-non-oil, RHA-O = RHA-oil, The four “Other” categories refer to non-oil lines, oil lines, open pollinated varieties and landraces, and introgressed lines, respectively.

Our STRUCTURE results using the full set of SNPs with MAF ≥10% indicated that *K* = 3 (hereafter referred to as Q = 3 corresponding to the Q matrix for the association testing results below), providing support for the existence of three genetically distinct clusters in our association panel. STRUCTURE results are grouped and graphed according to the line classifications (see Methods) in Figure 1. Delta*K* and the mean likelihood values are plotted in Figure S2. Clusters one and three largely consist of the maintainer (HA) lines whereas the majority of the restorer-oil (RHA-oil) lines exhibit substantial membership in cluster two. The PCoA analysis was largely consistent with the STRUCTURE results (Figure S3). In order to simplify the PCoA plot, we combined categories by grouping the lines into either HA, RHA-nonoil, RHA-oil, or other (this category contained all remaining lines/accessions). The RHA-oil lines are generally separated from the balance of the cultivated germplasm along the first and second axes, while the HA lines are generally distinct along the first axis.

Relative kinship was also estimated using the full set of markers (MAF ≥10%). Approximately 60% of the pairwise kinship estimates were near zero (i.e., less than 0.005), indicating the lines were essentially unrelated (Figure S4). The remaining estimates ranged from 0.05 to just less than 1, with a rapidly decreasing number of sunflower pairs exhibiting higher levels of relatedness.

### Genome-wide patterns of linkage disequilibrium and population structure

**Figure 2:**
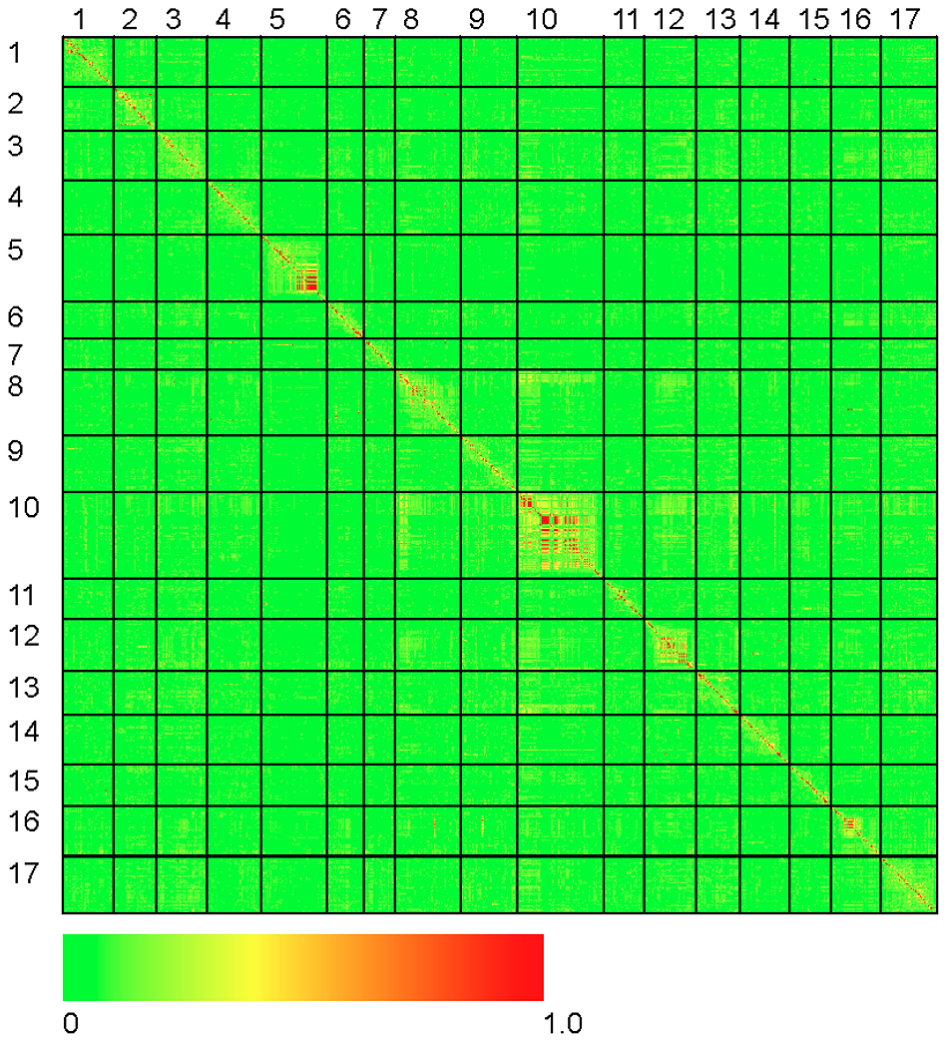
Heat map of linkage disequilibrium across the sunflower genome. Individual data points reflect squared allele frequency correlations (*r^2^*) for all possible pairs of polymorphic SNP markers, MAF ≥10%. The x- and y-axes correspond to the 17 linkage groups in sunflower with marker orders based on the work of Bowers et al. [21]. Note that the values above and below the diagonal are identical.

**Figure 3:**
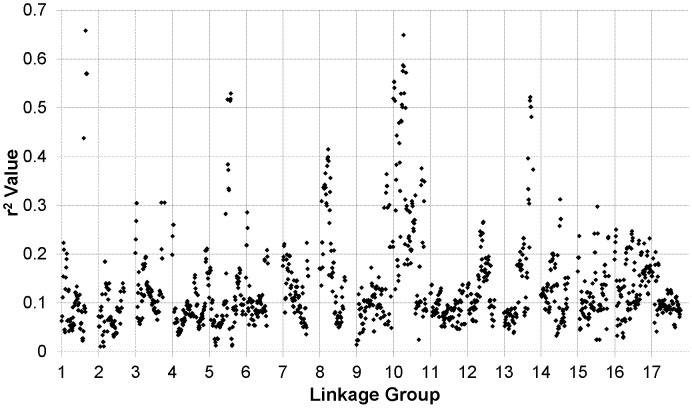
*r^2^* sliding window analysis. Sliding window analysis of squared allele frequency correlations (*r^2^*) across the sunflower genome.

The results of our genome-wide analysis of linkage disequilibrium (LD) are summarized in Figure 2 and Figure 3 and Figure S5. Figure 2 displays an LD matrix of the squared allele frequency correlations (*r* ^2^) plotted for the ordered markers (MAF ≥10%); the x- and y-axes correspond to the 17 LGs in sunflower. Regions of the genome where LD extends for considerable genetic map distance are visible as yellow to red squares on the figure (e.g., on LGs 5 and 10). Figure S5 shows plots of the squared allele frequency correlations (*r* ^2^) for 10,000 random pairs of SNPs within 50 cM as a function of genetic map distance between SNPs for the 17 LGs. Looking across chromosomes, different overall patterns of LD are apparent. For example, LG 10 exhibits a relatively slow overall decay in LD, largely due to strong haplotypic structure across a portion of the chromosome (see also Figure 2 and Figure 3), whereas LG 11 shows a much more rapid decay of LD. Following the methods of Macdonald et al. [31], the red line on each graph in Figure S5 summarizes the observed *r* ^2^ values as a function of map distance using the ksmooth function in the statistical software R (http://www.R-project.org/). On a per chromosome basis, the average genetic distance at which *r* ^2^ dropped below 0.1 ranged from 6.95 cM to 12.6 cM with the medians ranging from 3.93 cM to 10.1 cM. The sliding window analysis of *r* ^2^ further illustrates the variability in LD across the genome (Figure 3). In some cases, entire chromosomes show very low levels of LD. In other cases, elevated LD is visible in specific chromosomal regions, including portions of LGs 1, 5, 8, 10, and 13. The sliding window analysis of genetic diversity likewise revealed variation in UH_e_ across the genome (Figure S6).

**Figure 4:**
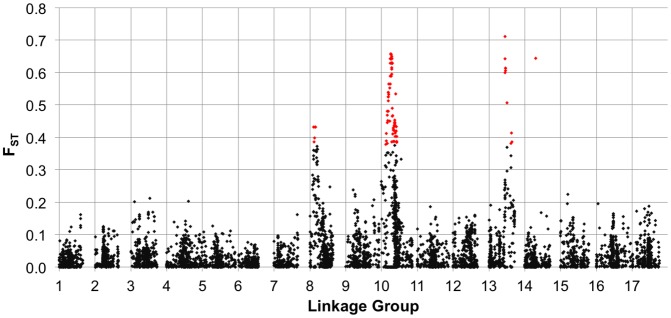
F_ST_ between heterotic groups. Population genetic differentiation (F_ST_) between heterotic groups (RHA vs. HA lines) for polymorphic SNPs, MAF ≥10%, plotted against genetic map position. The red colored dots represent individual SNPs that showed evidence of divergence due to selection in the outlier analysis.

We also estimated F_ST_ between the two primary breeding pools (i.e., RHA vs. HA lines) for the full set of markers (MAF ≥10%), plotted the results against genetic map position (Figure 4), and tested for selection using BayeScan. Genomic regions exhibiting significantly elevated differentiation are visible as spikes in F_ST_ (with individually significant markers being colored in red) on several chromosomes, including portions of LGs 8, 10, and 13, and a single marker on LG 14.

### Phenotypic diversity and association mapping

Our association mapping population exhibited substantial phenotypic diversity for both plant architecture and flowering time, expressed here in terms of branching and DTF. Significant positive correlations were found for both branching and flowering time across locations (i.e., more or less branched lines tended to behave similarly across locations, and the same was seen for DTF in terms of earlier vs. later flowering lines), whereas there was an overall significant negative correlation between branching and DTF (i.e., more highly branched plants tended to flower earlier; Figure S7). As noted above, there was a significant G×E interaction (*P*<0.01) for both traits studied. An important consequence of such G×E interactions is that different associations may be detected across environments; thus, we performed the association analyses separately for each location. The estimates of broad-sense heritability for branching and DTF were 0.861 and 0.124, respectively. The number of plants that showed a complete lack of branching was 79, 110, and 70 in Georgia, Iowa, and British Columbia respectively. The number of branches per genotype averaged 7.3, 7.2, and 7.6 and ranged from 1–29, 1–19, and 1–30 in Georgia, Iowa, and British Columbia respectively. DTF averaged 57.1 (range 41–77), 68.7 (45–95), and 80.4 (63–104) in Georgia, Iowa, and British Columbia respectively.

**Figure 5:**
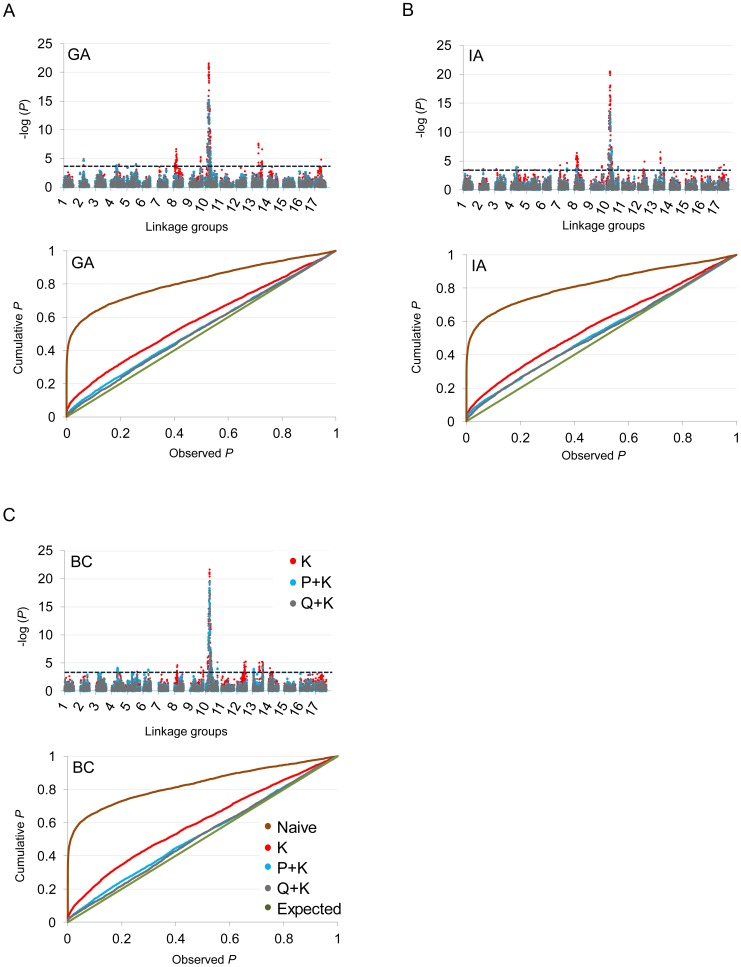
Manhattan and quantile–quantile plots of branching associations. (A, B, C) Upper panels: Manhattan plots of branching associations in three locations (GA, IA, BC respectively) plotted for the three models tested: red = K, blue = P+K, dark grey = Q3+K. The dashed line indicates the significance threshold based on the multiple testing correction method of Gao et al. [35] (alpha = 0.05, *P* = 0.00025, log 1/*P* = 3.60). Lower panels: Quantile-quantile plots of branching associations in all three locations plotted for the three models tested.

**Figure 6:**
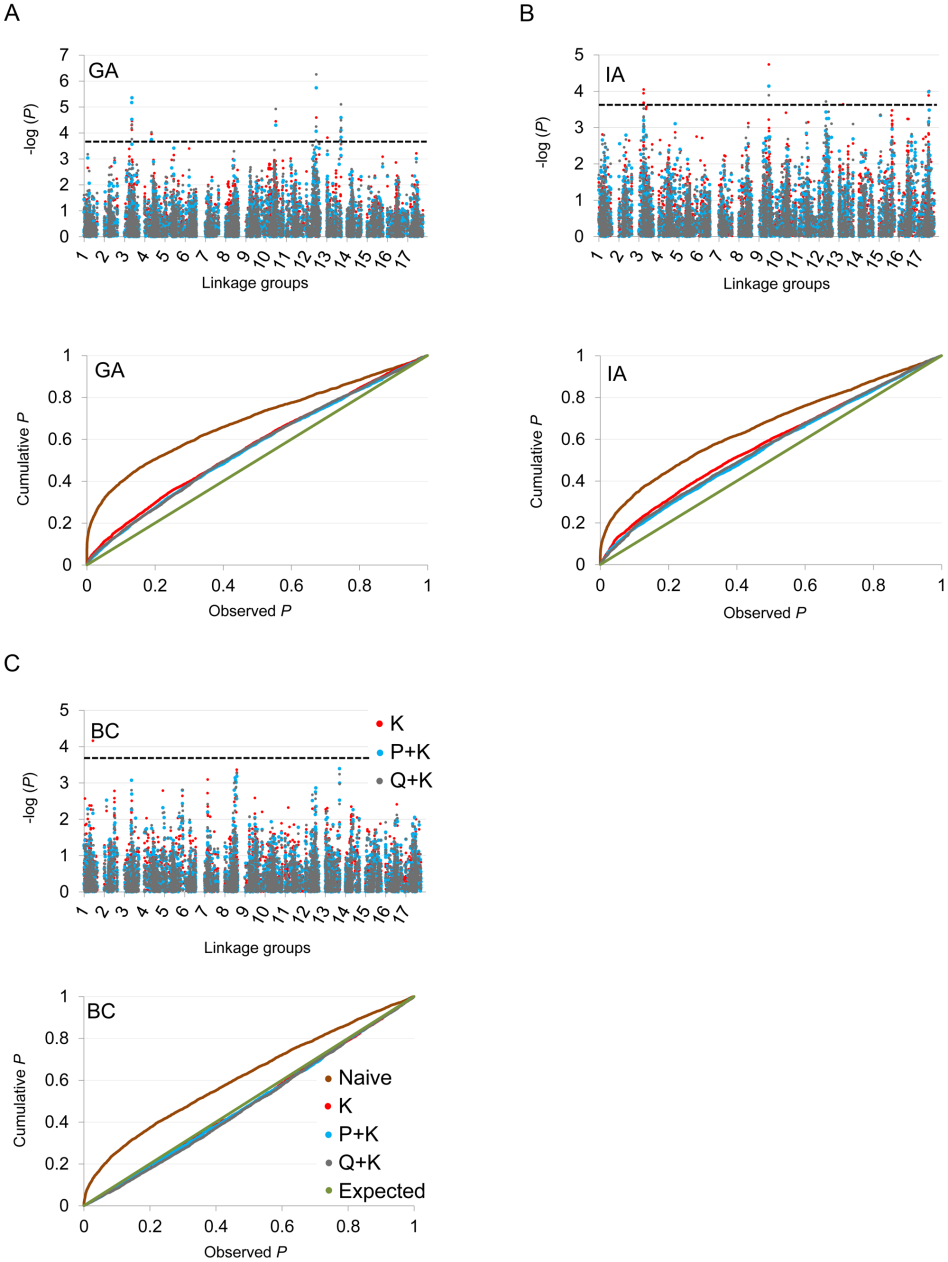
Manhattan and quantile–quantile plots of flowering time associations. (A, B, C) Upper panels: Manhattan plots of flowering (DTF) associations in three locations (GA, IA, BC respectively) plotted for the three models tested: red = K, blue = P+K, dark grey = Q3+K. The dashed line indicates the significance threshold based on the multiple testing correction method of Gao et al. [35] (alpha = 0.05, *P* = 0.00025, log 1/*P* = 3.60). Lower panels: Quantile-quantile plots of flowering time associations in all three locations plotted for the three models tested.

In terms of branching, our analyses revealed significant associations on LGs 2, 4, 5, 6, 7, 8, 9, 10, 12, 13, 14, and 17 (Figure 5; Table 2; Table S2). In general terms, each of these LGs had a single peak of significant marker-trait associations. The exceptions were: LG 7, which had two peaks in IA (one near 10 cM and another near 53 cM); LG 10, which had a primary peak centered near 25 cM in all locations and a secondary peak near 74 cM in IA and BC; LG 13, which had a primary peak near 41 cM and a secondary peak near 64 cM in all locations, as well as a third peak near 5 cM in BC; and LG 17, which had a primary peak near 41 cM in GA and IA and a secondary peak near 21 cM in IA. For DTF, our analyses revealed significant associations on LGs 1, 3, 4, 9, 10, 12, 13, and 17 (Figure 6; Table 3; Table S2). Here again, each of these LGs typically had a single peak of significant associations. The exception was LG 13, which had a primary peak near 70 cM in GA, a secondary peak near 3 cM in GA, and a third peak near 21 cM in IA. Table 2 and Table 3 summarize these results across locations for the three mixed models. Figure 5 and Figure 6 (upper panels) show the Manhattan plots for branching and DTF, respectively, in each of the three locations. For each location, the three mixed models are plotted as kinship (K, red), population structure as measured by PCA plus kinship (P+K, blue), and population structure as measured by STRUCTURE plus kinship (Q+K, dark grey). Points above the dashed threshold line are significant after correcting for multiple tests, as detailed above. Figure 5 and Figure 6 (lower panels) also show the quantile-quantile (q-q) plots of the observed *P-*values versus the expected for each of the three models as well as a naive model that does not account for population structure or kinship. As can be seen from the q-q plots, the distribution of observed *P-*values in the naive model greatly deviated from the expected distribution whereas the other models followed the expected distribution much more closely. This result reflects the potential confounding effects of population structure and relatedness in the dataset. Note, however, that for DTF in BC, two of the models (P+K and Q+K) provided fewer significant results than expected by chance, suggesting that these models may be overly conservative (e.g., [37]). The full set of results, including functional annotations for the genes from which the SNPs were derived (see [20]), significance values, and SNP effects for all individual loci at each location and using all three models, are provided in Table S2.

**Table 2:** Summary of significant branching associations.

Associations	GA			IA			BC		
	K	P+K	Q+K	K	P+K	Q+K	K	P+K	Q+K
**2**	x	x	x			x			
**4**	x	x		x	x			x	
**5**		x	x						
**6**								x	
**7a**				x					
**7b**				x	x				
**8**	x			x	x		x		
**9**	x			x			x		
**10a**	x	x	x	x	x	x	x	x	x
**10b**					x	x		x	x
**12**				x			x		
**13a**								x	
**13b**	x			x			x		
**13c**	x		x	x			x	x	x
**14**							x		
**17a**				x					
**17b**	x			x					

For each location, the results for three different models (K, P+K, and Q+K) are presented (see text for details). Associations are named based on their linkage group. When multiple associations were detected on a single linkage group (LGs 7, 10, 13, 17), the associations are lettered in order of their map position. Cases in which more than one model within a location supported an association are underlined.

**Table 3:** Summary of significant flowering time (DTF) associations.

Associations	GA			IA			BC		
	K	P+K	Q+K	K	P+K	Q+K	K	P+K	Q+K
**1**							x		
**3**	x	x	x						
**4**		x	x						
**9**					x	x			
**10**	x	x	x						
**12**	x	x	x			x			
**13a**	x								
**13b**				x					
**13c**	x	x	x						
**17**				x	x				

For each location, the results for three different models (K, P+K, and Q+K) are presented (see text for details). Associations are named based on their linkage group. When multiple associations were detected on a single linkage group (LG 13), the associations are lettered in order of their map position. Cases in which more than one model within a location supported an association are underlined.

**Figure 7:**
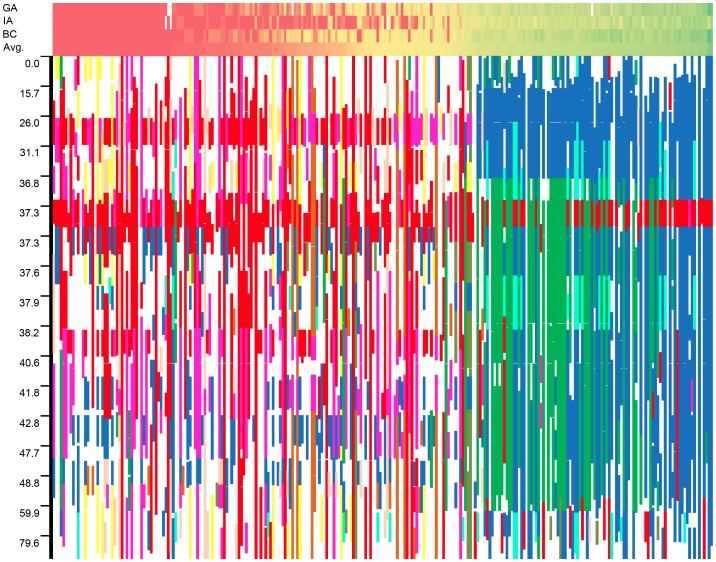
Graphical genotypes from linkage group 10. Graphical genotypes from linkage group 10 plotted against a heat map of the branching data sorted by average level of branching across all three locations. Unbranched plants (red) are to the left whereas highly branched plants (green) are to the right. Note that the scale of the y-axis scale changes based on marker density, and white squares in the phenotype heat map represent missing data in an individual location.

The graphical genotypes for all 17 LGs across the full population of 271 accessions are presented in Figure S8. The genotype with the highest fraction of shared haplotypes across the genomes of the 271 lines (G1 in red) corresponds to the accession HA89, a line of great historical importance in sunflower breeding. HA89 accounted for an average of 16.2% of the genomes of the 271 lines. Overall, the 25 most common genotypes accounted for an average of 63.5% of the genomes of the 271 lines examined. For ease of interpretation, only the top nine genotypes are color-coded (beyond this point, each additional genotype individually accounted for ca. 1% or less of the genome); in total, these nine genotypes accounted for an overall average of 50.7% of the genomes of the 271 lines (see Table S3). White regions either correspond to non-major genotypes or reflect stretches with fewer than 25 consecutive, homozygous SNPs. Figure 7 depicts the results for LG 10 with the data sorted by the average number of branches produced by plants of each accession at all three locations (see heat map along the top). As noted above, the upper portion of this LG exhibits strong haplotypic structure and elevated LD across an extended region along with a major effect on branching at all three locations. See below for a detailed discussion of the historical and biological significance of these results.

## Discussion

The work presented herein represents the largest and most comprehensive analysis of population genomic diversity in cultivated sunflower to date. In terms of overall levels of SNP diversity, our data indicate that sunflower exhibits considerable molecular variation, on par with estimates derived from large-scale SNP surveys of other crops (e.g., maize [38]–[40], barley [41], and rice [42]). We also documented substantial phenotypic variation in terms of both plant architecture and flowering time, ranging from a complete lack of branching to whole plant branching and including accessions that reached reproductive maturity over a period spanning greater than 30 days. Thus, despite the population genetic bottlenecks that are known to have occurred during domestication and improvement, the cultivated sunflower gene pool harbors substantial variability.

In terms of overall population structure, our results are largely in agreement with our prior analyses based on a much smaller set of simple-sequence repeat markers (SSRs) [22]. This general agreement between our current findings and the earlier, SSR-based work suggests that any possible ascertainment bias during SNP discovery and selection had minimal effects on our population genetic results. In fact, the preferential usage of SNPs with high heterozygosity would be expected to result in an underestimate of the magnitude of structure [43] but the F_ST_ estimates between the B and R lines is virtually identical between the SNPs (F_ST_ = 0.049) and the SSRs (F_ST_ = 0.047). The much larger number of markers in the present study has, however, allowed us to refine our earlier findings. Notably, we found evidence for the presence of three genetically distinct groups within the germplasm collection. One of these groups was primarily composed of the RHA lines, while a second group consisted of a large number of HA lines, and the third included a more diverse assemblage of lines. The PCoA likewise demonstrated a split between the RHA and HA lines, with an even clearer division between the RHA-oil and HA lines. This genetic distinction between B and R lines is expected given the breeding history of sunflower, which involves the maintenance of distinct gene pools to maximize heterosis in hybrid crosses [44], [45]. Taken together, these results underscore the need to account for population structure when performing association analyses in sunflower.

Our analysis of LD revealed considerable variability across the genome. In most regions, LD declined quite rapidly as a function of genetic distance and the correlation between most pairs of SNPs fell to negligible levels (i.e., *r^2^*≤0.10) within 3 cM. In some instances, however, LD remained elevated (on average) over greater distances (e.g., LG 10; see Figure S5). Inspection of Figure 2 and Figure 3 reveals the existence of a number of localized islands of LD, including blocks on LGs 1, 5, 8, 10, 13, 16, and 17. In looking more closely at these localized regions exhibiting elevated LD, it is apparent that many of them occur in close proximity to genes or QTL underlying traits that have been targeted by selection during sunflower domestication and/or improvement. Given the history of breeding for resistance to diseases in sunflower [46] it is noteworthy that the four of these spikes in LD (on LGs 1, 5, 8, and 13) co-localize with QTL and/or candidate genes for resistance to several important diseases. Note that co-localization of QTL and LD spikes/associations (below) is based upon concordance of shared genetic markers (most often microsatellites) between the previous QTL map(s) and the map of Bowers et al. [21]. More specifically, QTL and/or genes for resistance to downy mildew (*Plasmopara halstedii*; [47]–[52]) co-localize with spikes in LD on LGs 1 and 8. Similarly, the block of elevated LD observed on LG 5 co-localizes with a QTL for resistance to black stem (*Phoma macdonaldii*, [53]). Finally, the spike in LD on LG 13 co-localizes with sunflower rust resistance genes [50], [52], [54]. It thus appears that selection for disease resistance may have played a role in shaping genome-wide patterns of LD in sunflower, though we cannot rule out the possibility of selection on other traits (see below).

The important role that selection on plant architecture played during the domestication and subsequent improvement of sunflower also appears to have shaped patterns of genetic diversity across the sunflower genome, especially with respect to LG 10. During the initial domestication of sunflower, unbranched, monocephalic landraces were preferentially propagated and the domesticated lineage moved away from the intensely branched, multi-headed phenotype that is characteristic of wild sunflower [17], [55]–[57]. Until the mid-20^th^ century, modern cultivars were thus typically unbranched; however, beginning in the late 1960s, the transition to hybrid breeding and the associated desire for a prolonged flowering period in male lines resulted in the re-introduction of branching into the sunflower gene pool [45]. This resulted in selection favoring the fixation of a recessive branching allele at the so-called *B*-locus in R lines. The *B*-locus has since been mapped to approximately 27 cM from the top of LG 10 [58].

In viewing the graphical genotypes, the *B*-locus is visible as differentiated haplotypic blocks that span this region on LG 10 and which clearly correlate with the extent of branching (Figure 7). Interestingly, the re-introduced branching haplotype (in dark blue) spans ca. 25 cM, whereas the unbranched haplotype (primarily in red) appears to span ca. 10 cM. Thus, the effects of the very recent re-introduction of branching to the cultivated gene pool can be visualized as a large haplotypic block presumably resulting from a recent, strong selective sweep in the branched R (RHA) lines. This pattern can also be seen in the sliding window analyses of LD (*r* ^2^) and the plots of population differentiation (F_ST_) vs. genetic map position. In both cases, spikes are clearly visible in that same region along LG 10 (Figure 3 and Figure 4). Notably, our BayeScan analysis indicated that this region of the genome, along with portions of LG 8 and 13 and a single marker on LG 14, exhibits significantly elevated F_ST_ (Figure 4). This finding is consistent with the notion that this differentiation – which spans a remarkably long (at least in genetic terms) and otherwise recombinogenic genomic interval – was driven by strong selection.

Not surprisingly, this same region of LG 10 harbored highly significant associations for branching in all three locations, as well as for DTF in GA. Overall, we found significant associations for branching in 17 genomic regions on 12 of the 17 LGs in sunflower. In five cases, these associations overlapped with previously identified QTL for branching in sunflower on LGs 10 (a), 12, 13 (a and b) and 17 (Figure S9) [9], [59]–[61]; the remainder were novel effects for number of branches that have not previously been documented. Similarly, we found significant associations for DTF in 10 genomic regions located on 8 of the 17 sunflower LGs (Figure S10) [9], [62]; the remainder (on LGs 1, 3, 4, 10, 12, 13) were novel effects. It is noteworthy that the detected associations generally spanned much smaller intervals than are typical of traditional QTL studies. This result is consistent with our finding that LD decays relatively rapidly across much of the genome (see above) and suggests that even higher marker densities would be desirable for developing a complete picture of trait variation in sunflower.

In terms of the relationship between marker-trait associations and both LD and population differentiation, the branching and DTF associations co-localized with spikes in *r* ^2^ and F_ST_ (on LGs 8, 10, and 13; Figure 3 and Figure 4). As noted above, all three of these regions, along with a single marker on LG 14, exhibited significantly elevated F_ST_ values, suggestive of selective divergence. Given the historical importance of the *B*-locus and the clear correspondence of the observed haplotypes to sunflower breeding groups (and thus branching architecture; see above), it seems likely that the driving force behind the pattern observed on LG 10 was selection on branching. The situation on LGs 8 and 13 is less clear; the observed patterns may have been driven be selection on branching, flowering time, disease resistance, or some combination thereof. It must be kept in mind, however, that the branching associations that we detected on LG 8 were most apparent in the kinship-only model, and disappeared almost entirely when we controlled for population structure. As such, this result in particular may have been a byproduct of population structure as opposed to a true functional association.

It is particularly noteworthy that the majority of significant associations identified herein are located in the aforementioned islands of LD. Given that our analyses relied on a single SNP in each of ca. 5,300 genes, we are almost certainly missing out on associations in regions of low LD. In fact, nearly 50% of the sliding windows analyzed had an average *r* ^2^<0.1 and over 85% had an average *r* ^2^<0.2. As such, future analyses aimed at assaying genetic variation at a much higher density (e.g., using genotyping-by-sequencing [63] or even whole genome re-sequencing) seem warranted and are likely to facilitate a much more detailed characterization of the molecular basis of phenotypic variation in sunflower.

## Supporting Information

Figure S1 Map of the locations of the three field sites. Georgia (GA), United States; Iowa (IA), United States; and British Columbia (BC), Canada. Map from d-maps.com.
(PDF)

Figure S2 Summary of STRUCTURE results. The plots of the Delta*K* and log-likelihood values for the STRUCTURE analyses.
(PDF)

Figure S3 Principal coordinates analysis (PCoA) using all polymorphic SNP markers. Line classifications were simplified to HA, Other, RHA, and RHA-Oil in order to improve viewing of the figure. See text for details.
(PDF)

Figure S4 Plot of the frequency and distribution of kinship, or pairwise relatedness, amongst accessions in the association population. Values of 0.5 or greater were grouped into a single category.
(PDF)

Figure S5 Linkage group plots of the squared allele frequency correlations (*r^2^*). Plots of the squared allele frequency correlations (*r^2^*) as a function of genetic map distance between 10,000 randomly selected pairs of SNPs separated by 50 cM or less. The red line on each graph summarizes the observed *r* ^2^ values as a function of map distance using the ksmooth function in the statistical programming language R (see text for details).
(PDF)

Figure S6 Sliding window analysis of genetic diversity. Sliding window analysis of the unbiased expected heterozygosity (UH_E_) across the sunflower genome.
(PDF)

Figure S7 Correlation matrix for the branching and flowering time (DTF) data across the three locations. The cells are color coded to indicate positive (red) or negative (blue) correlations. Significant correlations after correcting for multiple tests are starred (see text for details).
(PDF)

Figure S8 Graphical genotypes for the 17 LGs across the full population of 271 accessions. Accessions were grouped according to the previously defined accession categories as in Figure 1. The top nine genotypes were color-coded (see Table S3 for details of coding). White regions are either non-major haplotypes or regions with fewer than 25 consecutive, homozygous SNPs.
(PDF)

Figure S9 Co-localization of branching associations and QTL. Comparison of genetic map positions for significant associations and previously identified branching QTL.
(PDF)

Figure S10 Co-localization of flowering time associations and QTL. Comparison of genetic map positions for significant associations and previously identified flowering time QTL.
(PDF)

Table S1 Association population with class and USDA designation.
(XLSX)

Table S2 Association mapping results for branching in the three locations. Note that, because the sign of the effect estimates is a byproduct of arbitrary allele assignments, these data are presented as absolute values.
(XLSX)

Table S3 Reference individuals for graphical genotypes.
(XLS)
