# Supplementary figures and images for: Association Mapping and the Genomic Consequences of Selection in Sunflower
Source: PLoS Genet. 2013 Mar 21;9(3):e1003378. doi: 10.1371/journal.pgen.1003378 (PMC3605098; doi:10.1371/journal.pgen.1003378)

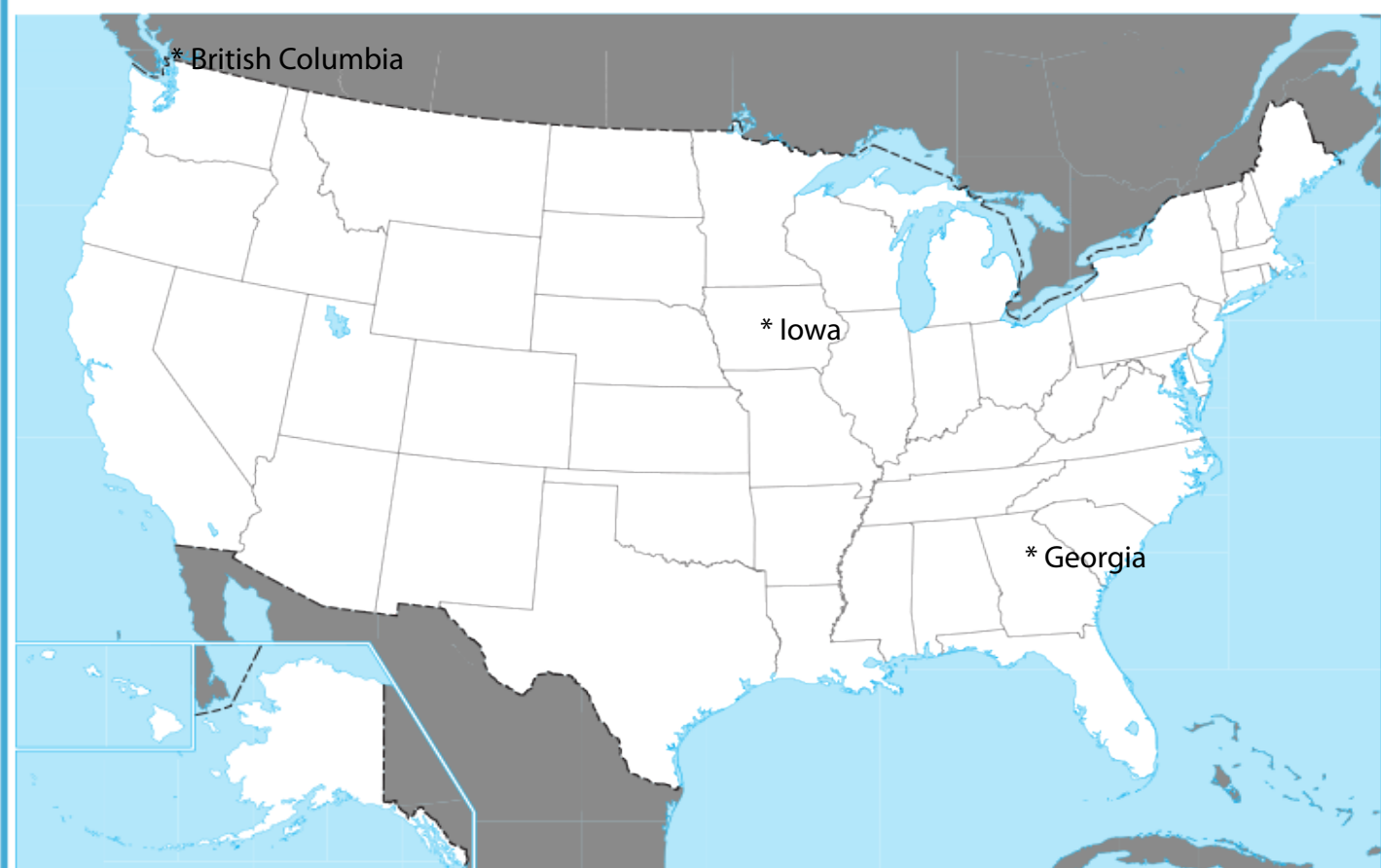

Supplement: Figure S1 — Map of the locations of the three field sites. Georgia (GA), United States; Iowa (IA), United States; and British Columbia (BC), Canada. Map from d-maps.com. (PDF) [file pgen.1003378.s001.pdf]

$$\text{DeltaK} = \text{mean}(|L''(K)|) / \text{sd}(L(K))$$

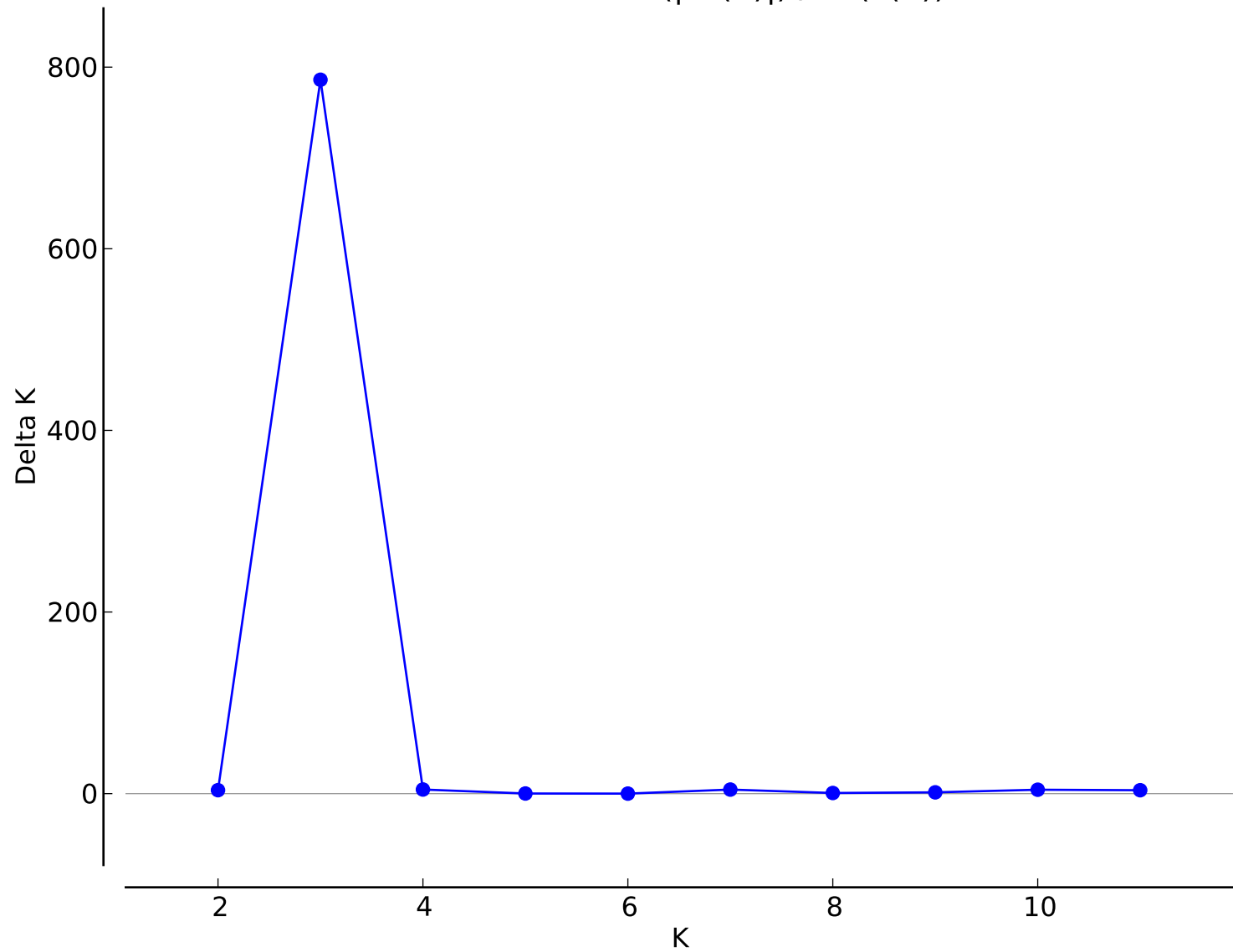

L(K) (mean  $\pm$  SD)

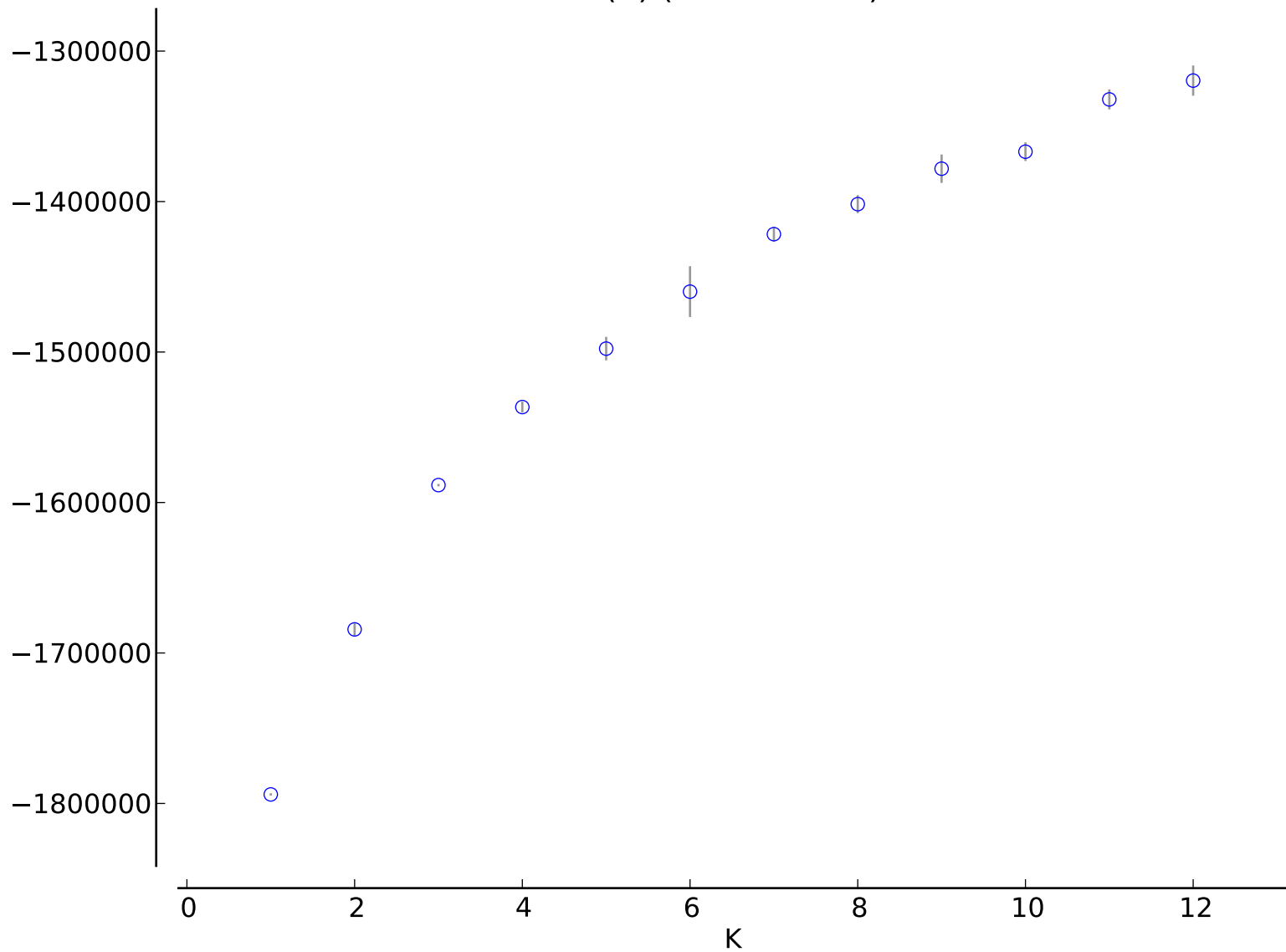

Supplement: Figure S2 — Summary of STRUCTURE results. The plots of the DeltaK and log-likelihood values for the STRUCTURE analyses. (PDF) [file pgen.1003378.s002.pdf]

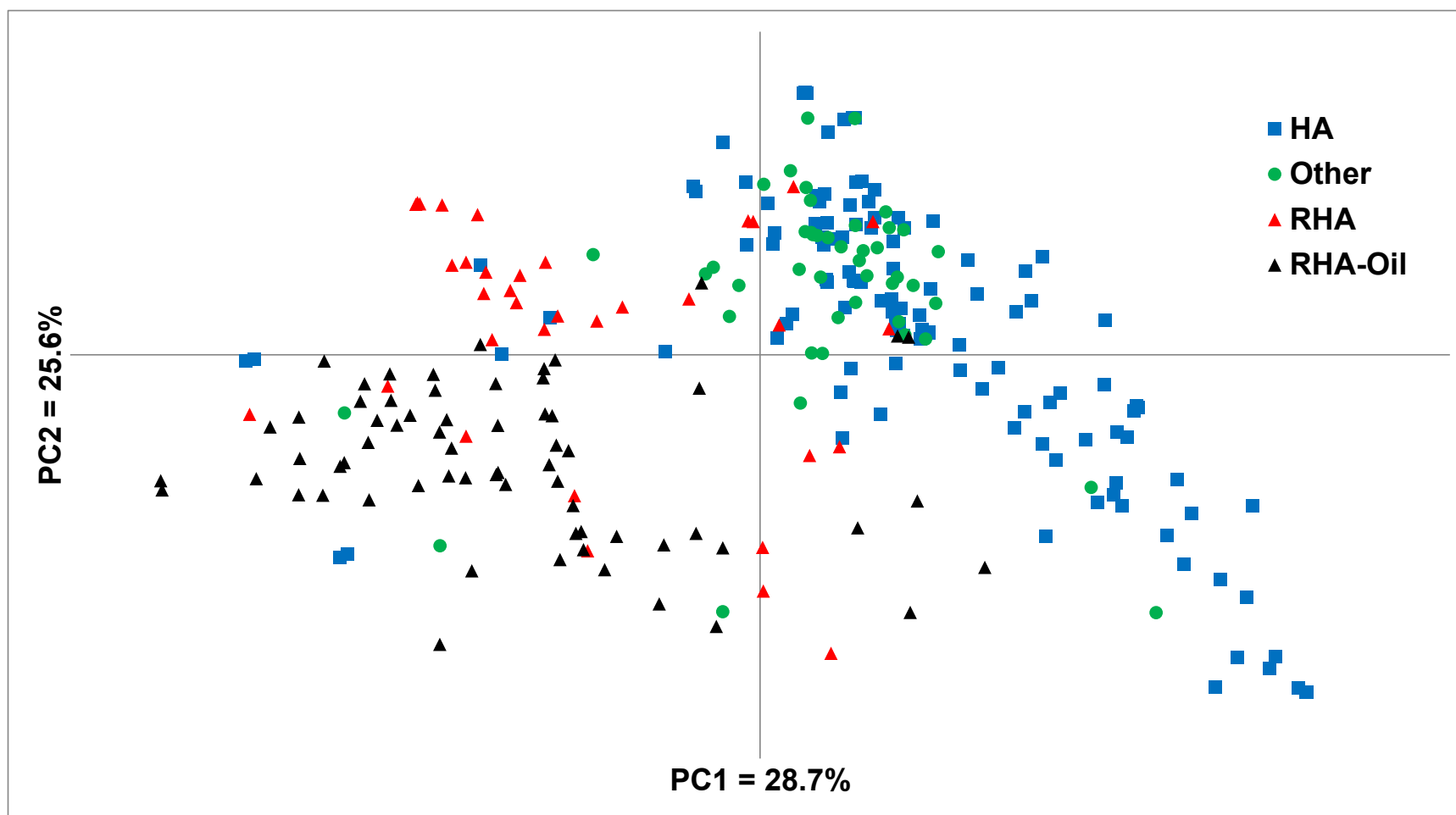

Supplement: Figure S3 — Principal coordinates analysis (PCoA) using all polymorphic SNP markers. Line classifications were simplified to HA, Other, RHA, and RHA-Oil in order to improve viewing of the figure. See text for details. (PDF) [file pgen.1003378.s003.pdf]

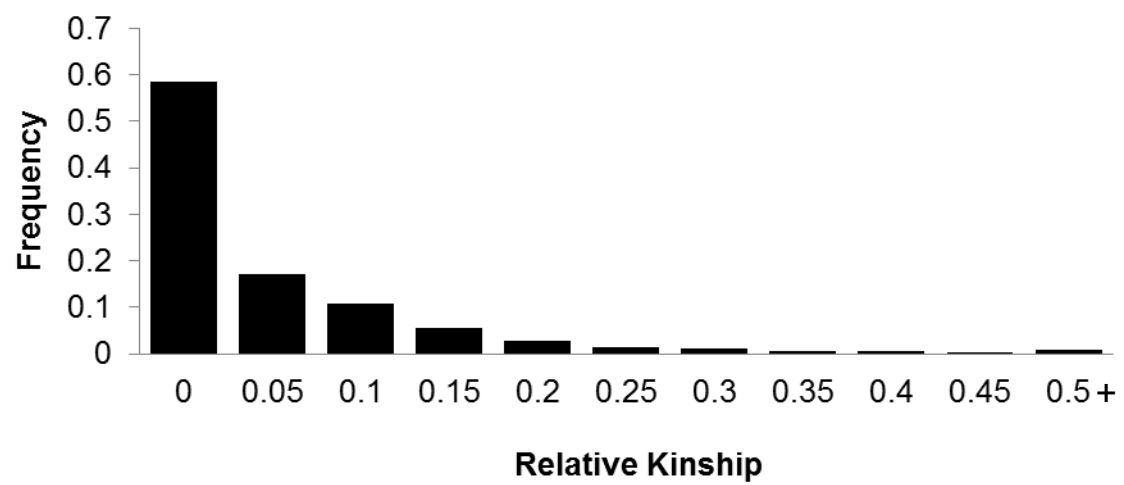

Supplement: Figure S4 — Plot of the frequency and distribution of kinship, or pairwise relatedness, amongst accessions in the association population. Values of 0.5 or greater were grouped into a single category. (PDF) [file pgen.1003378.s004.pdf]

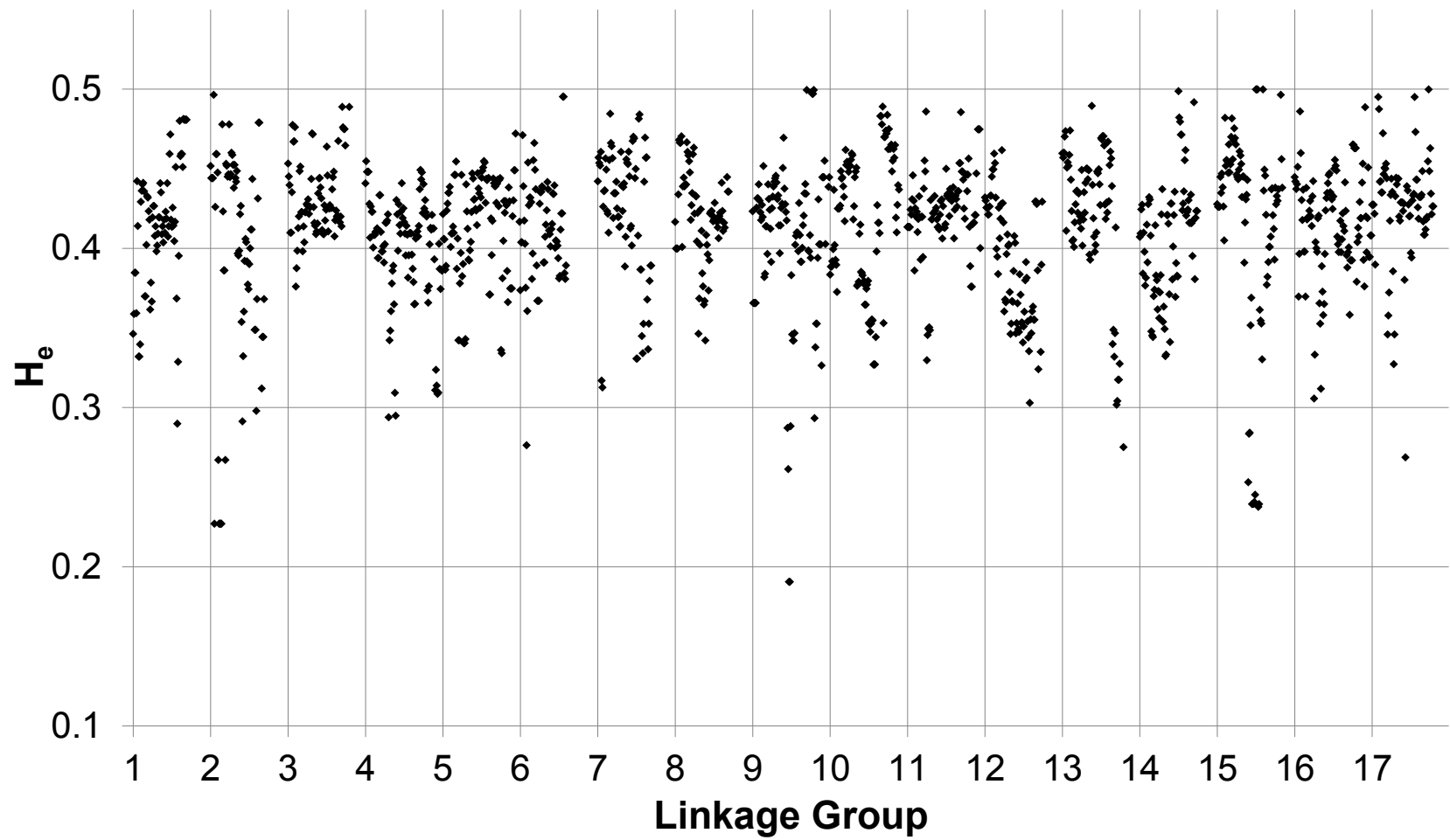

Supplement: Figure S6 — Sliding window analysis of genetic diversity. Sliding window analysis of the unbiased expected heterozygosity (UHE) across the sunflower genome. (PDF) [file pgen.1003378.s006.pdf]

LG 1

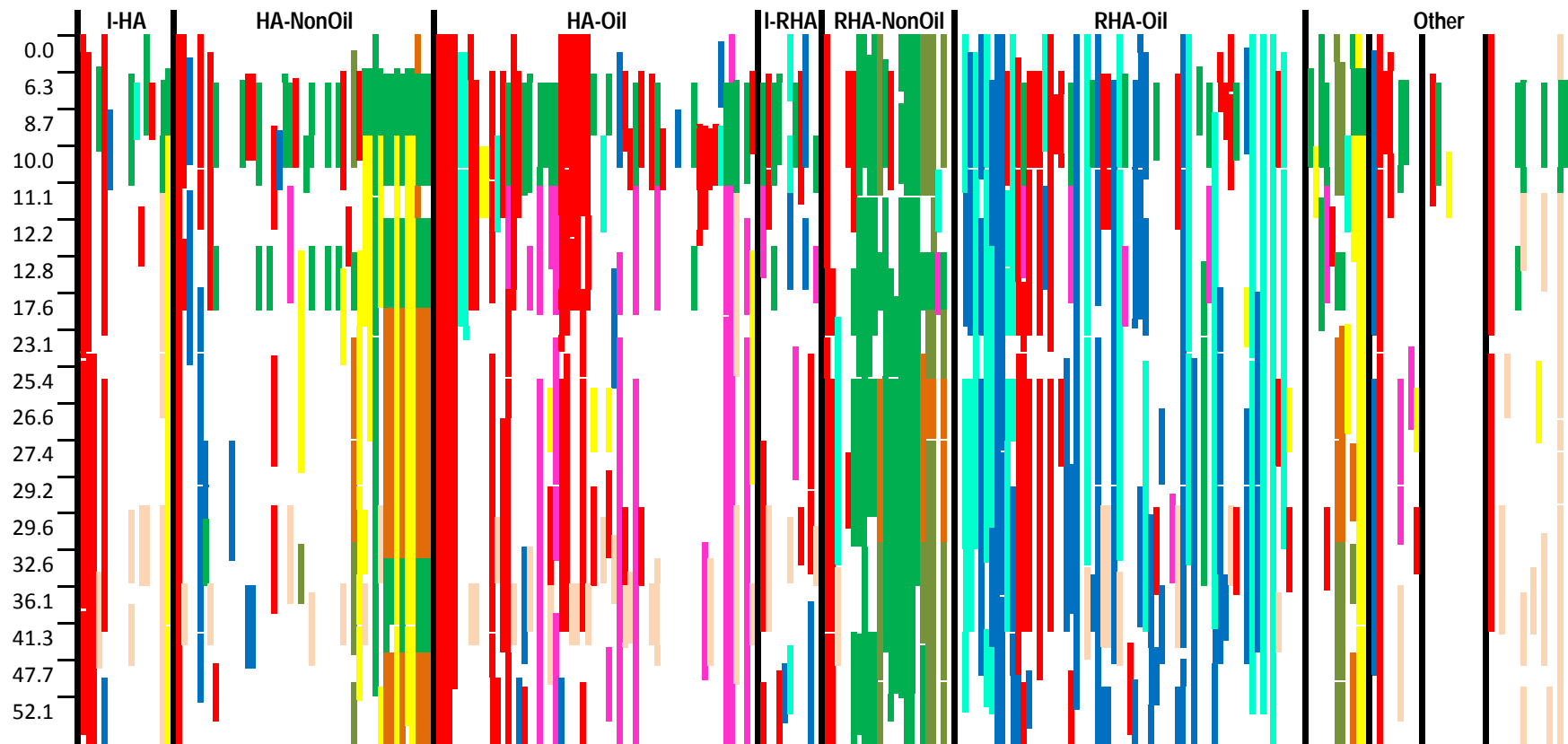

LG 2

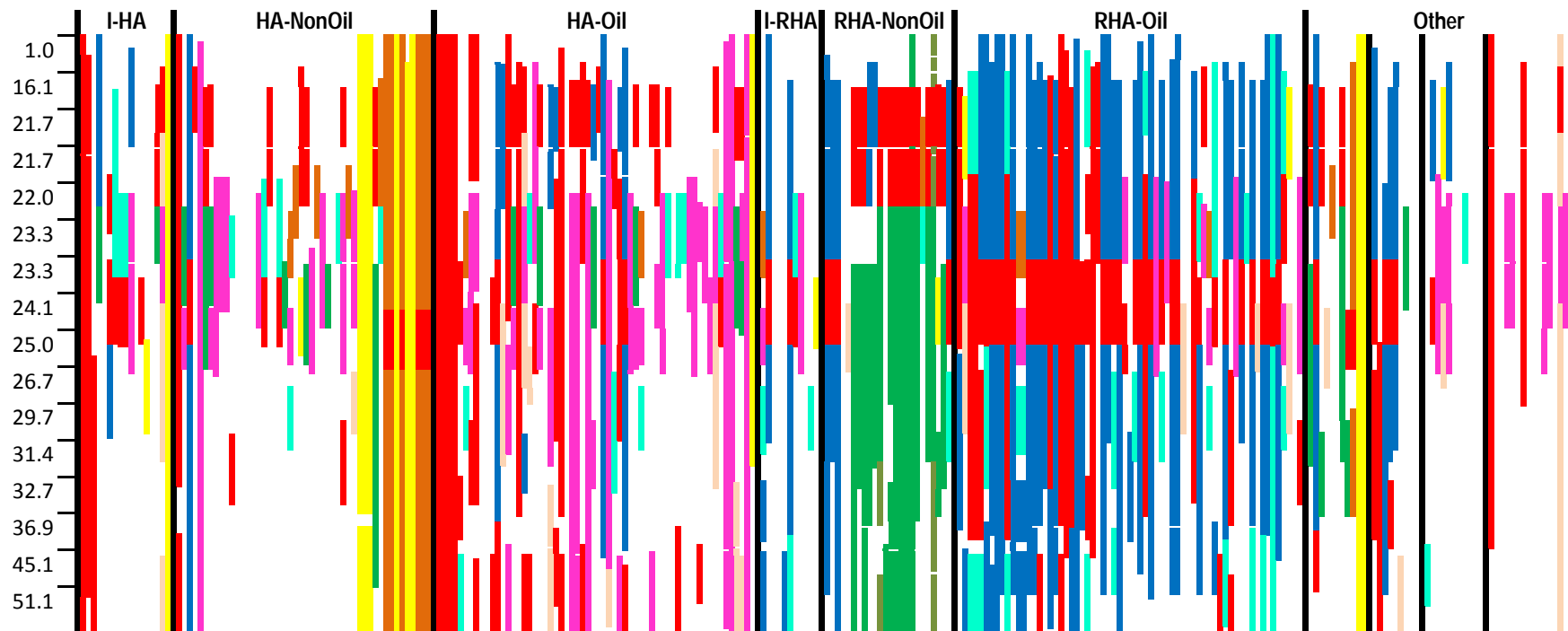

LG 3

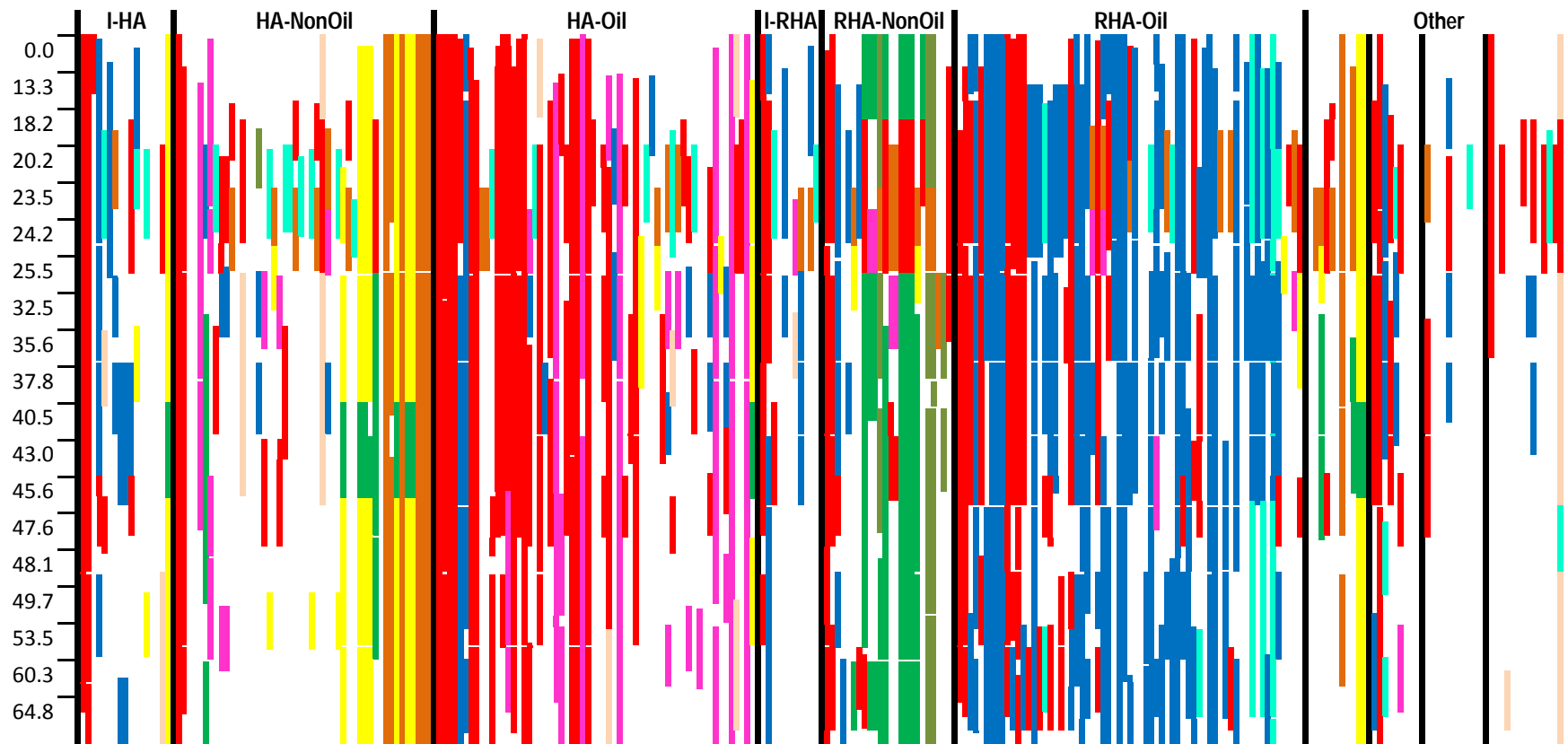

LG 4

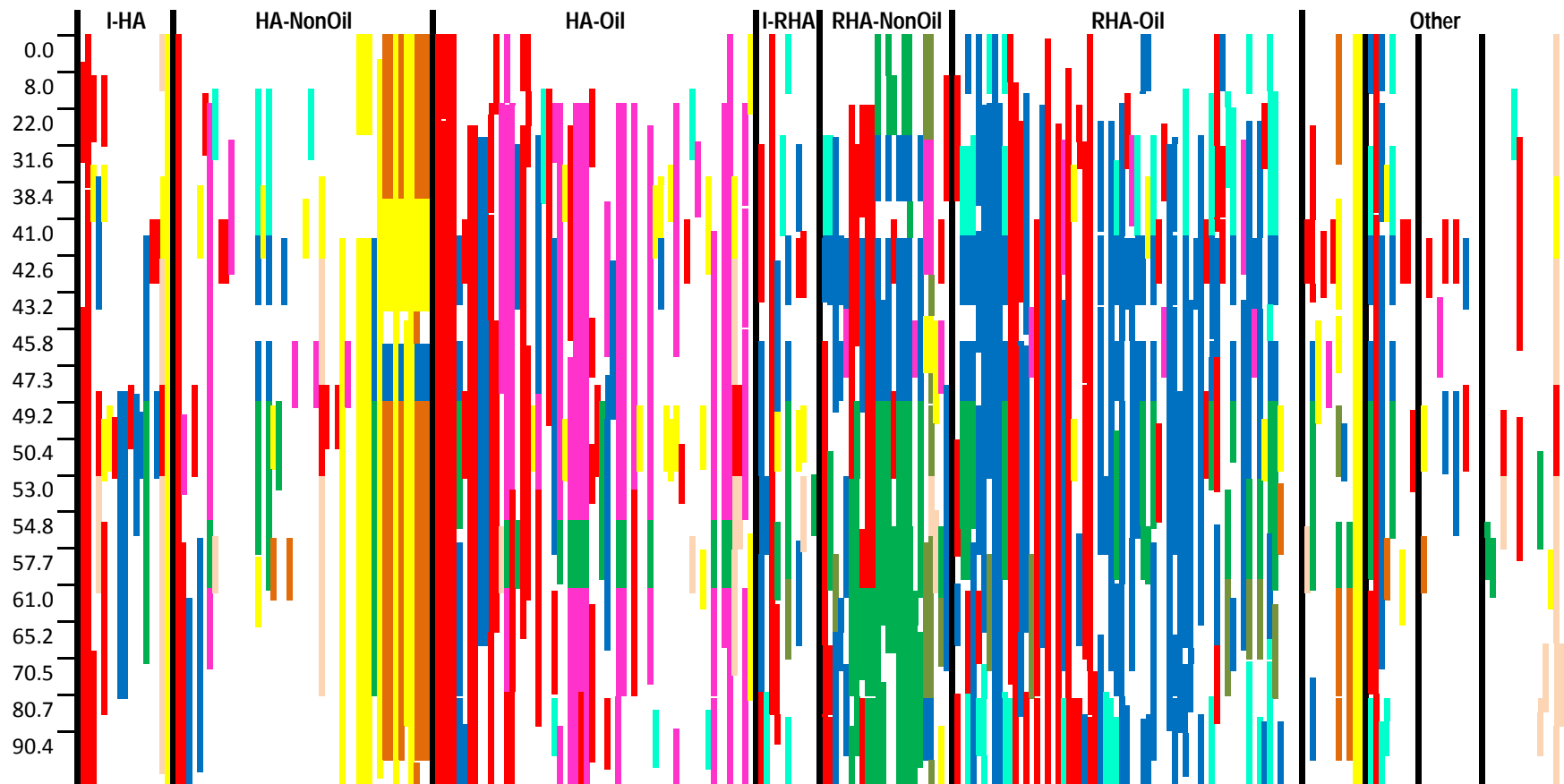

LG 5

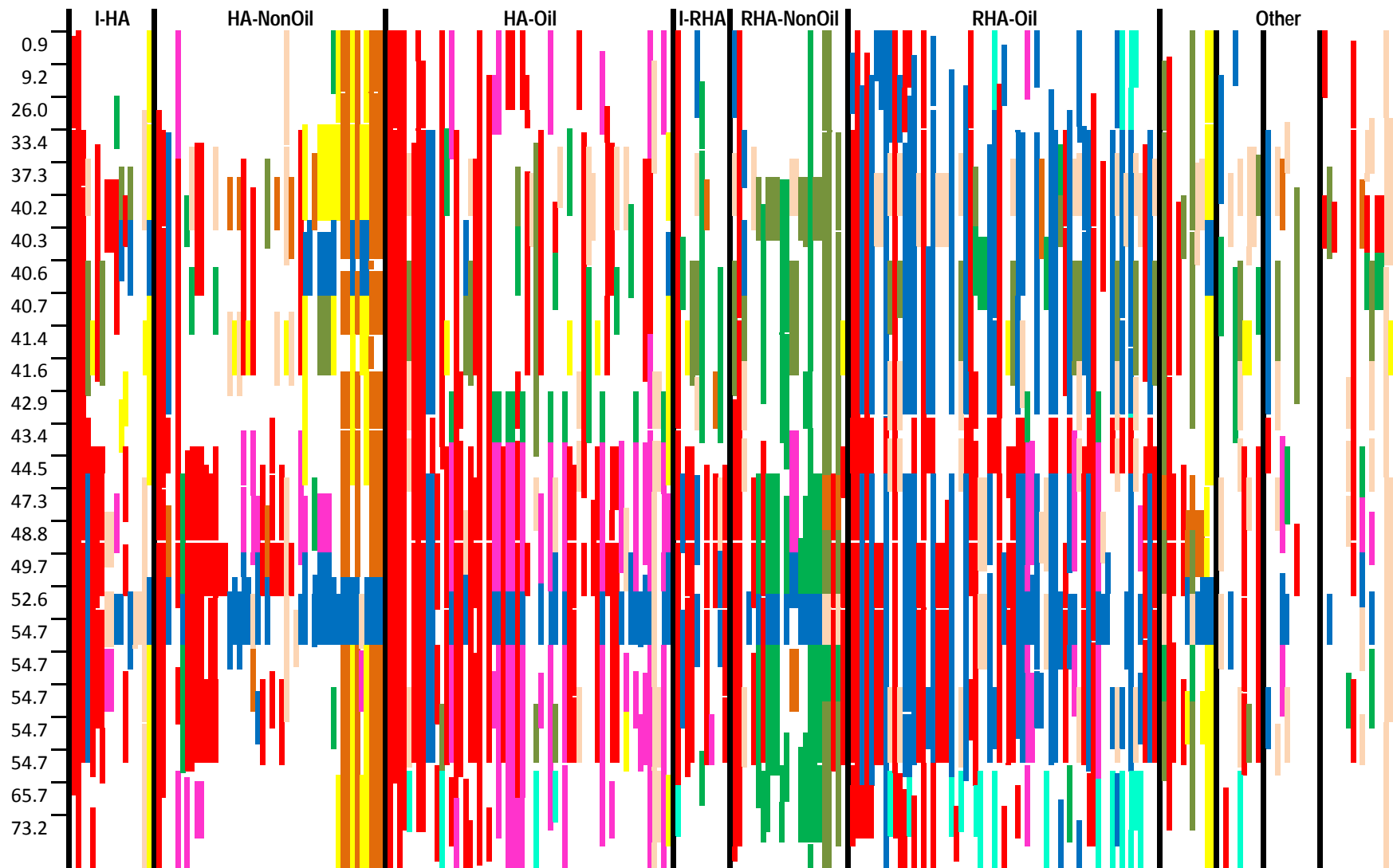

LG 6

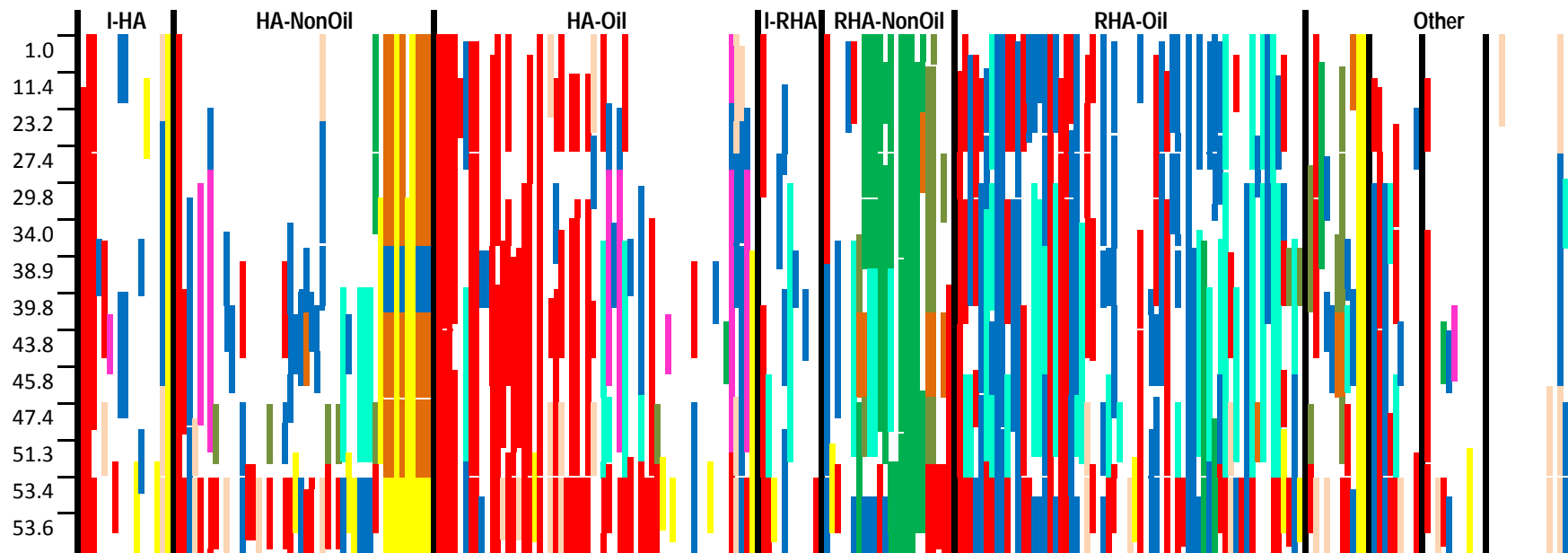

LG 7

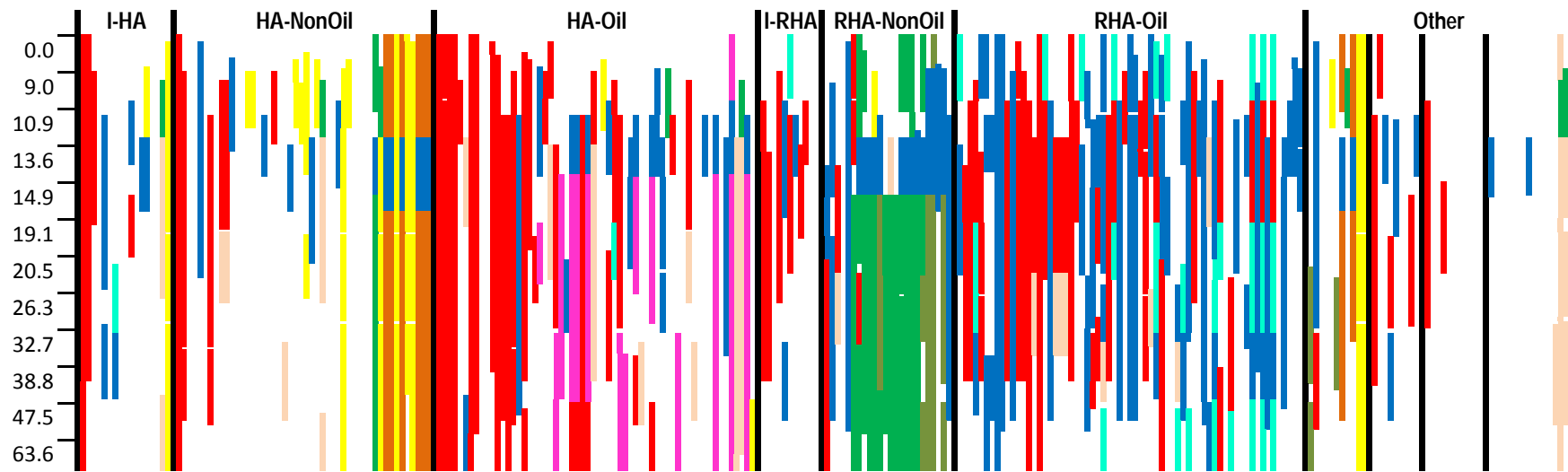

**LG 8**

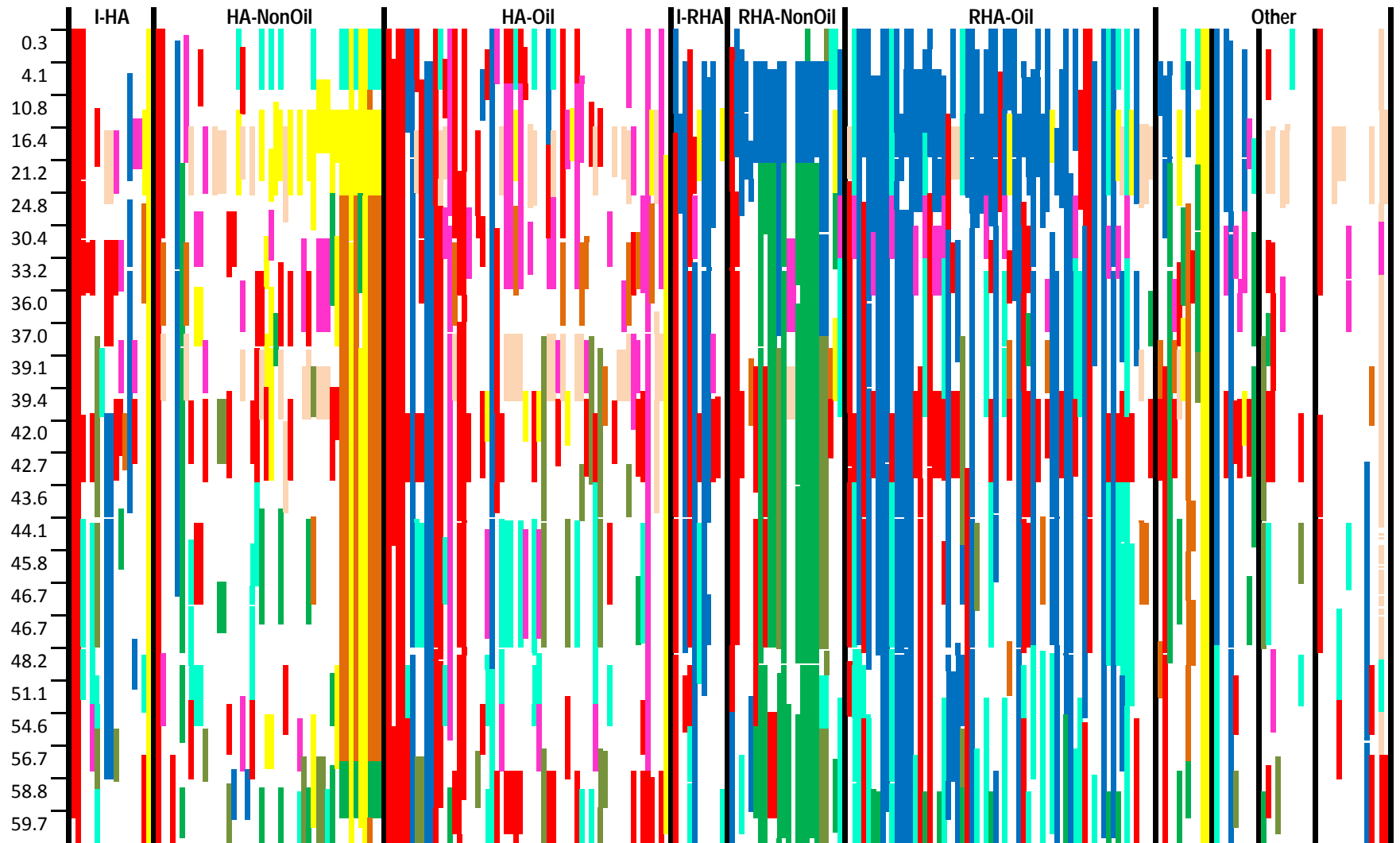

LG 9

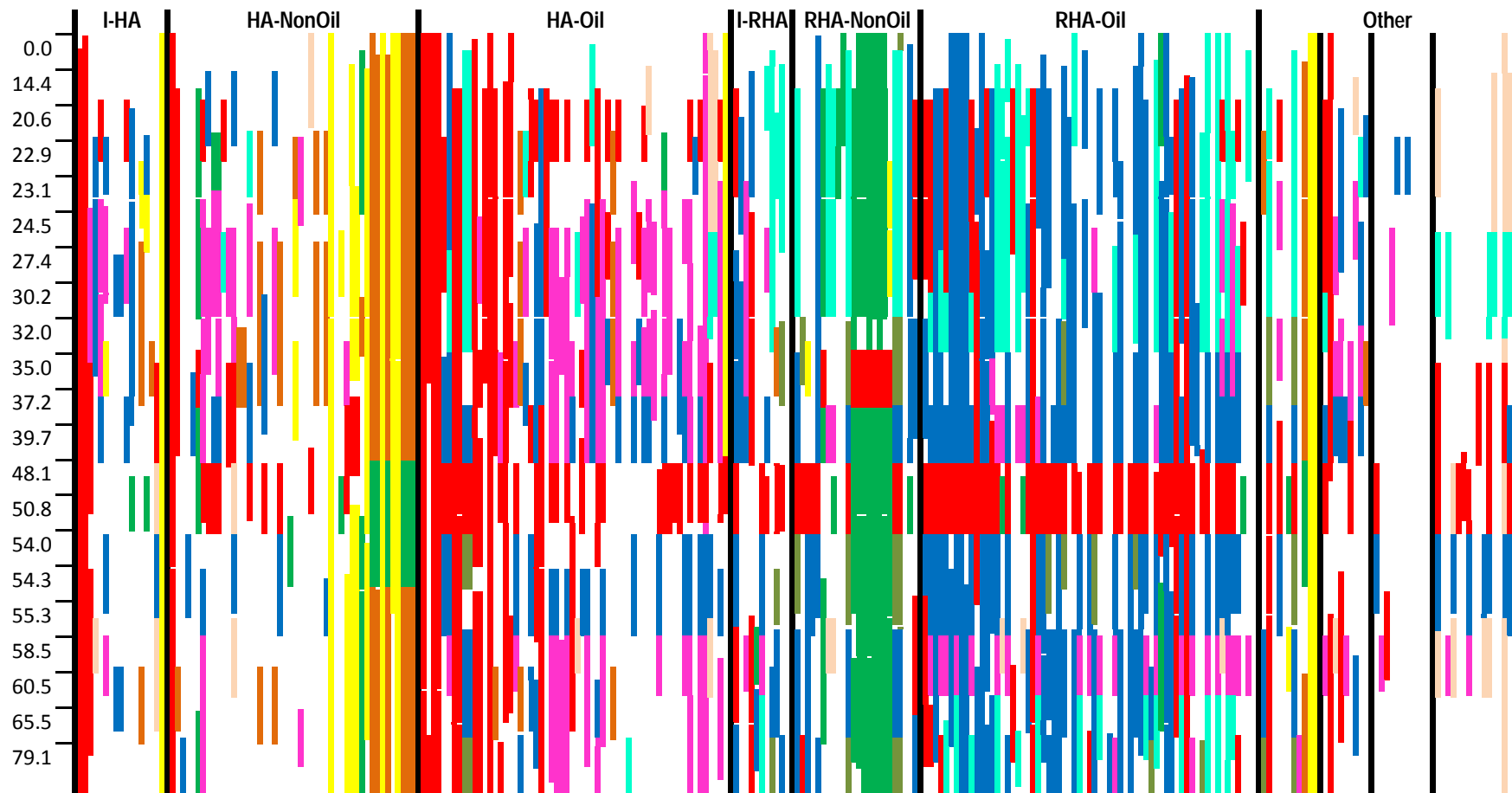

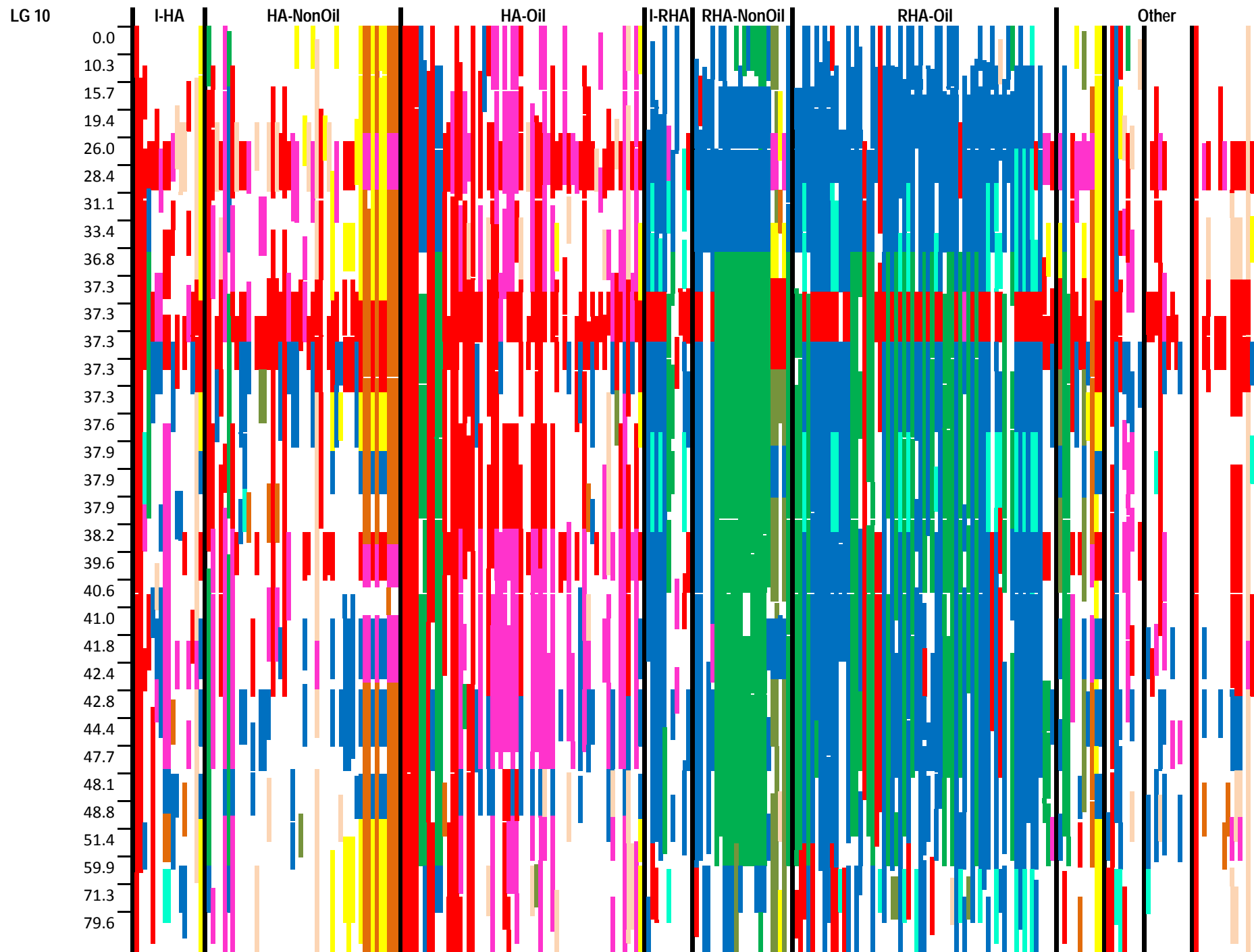

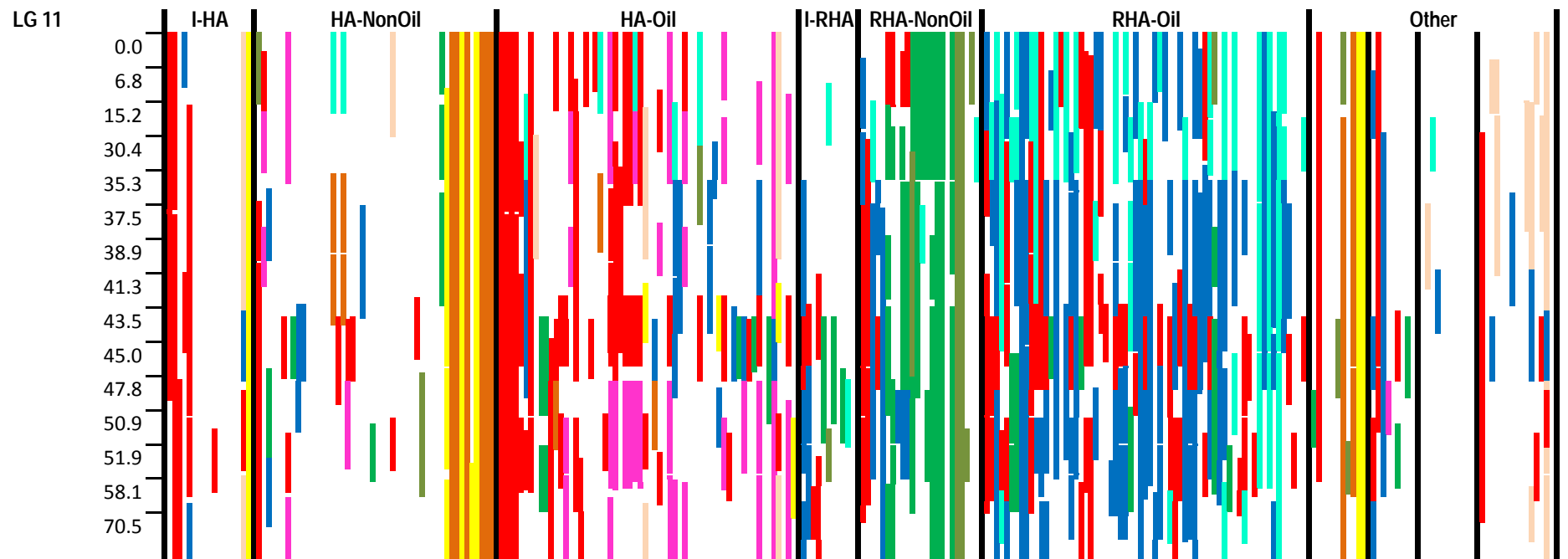

LG 12

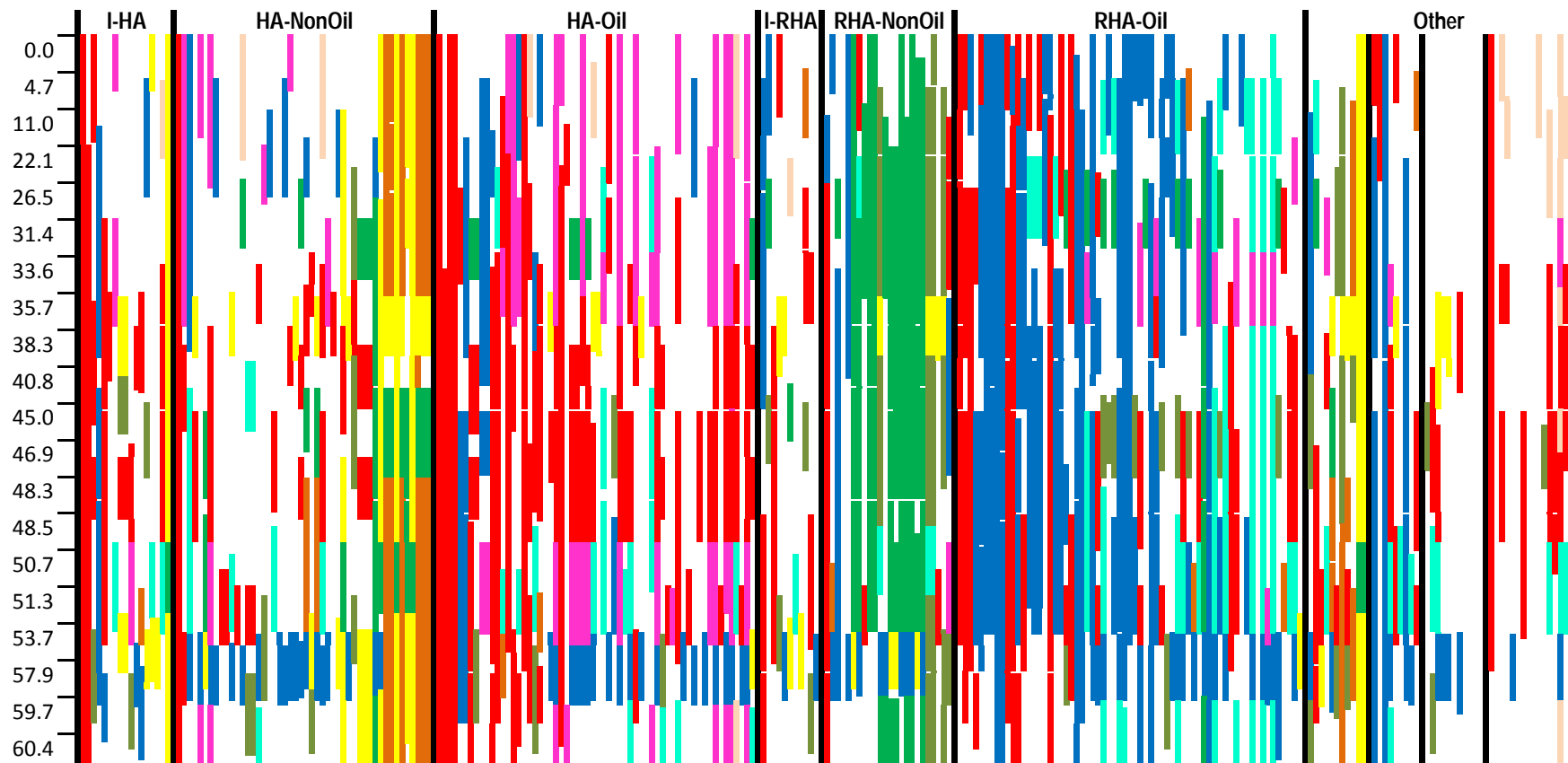

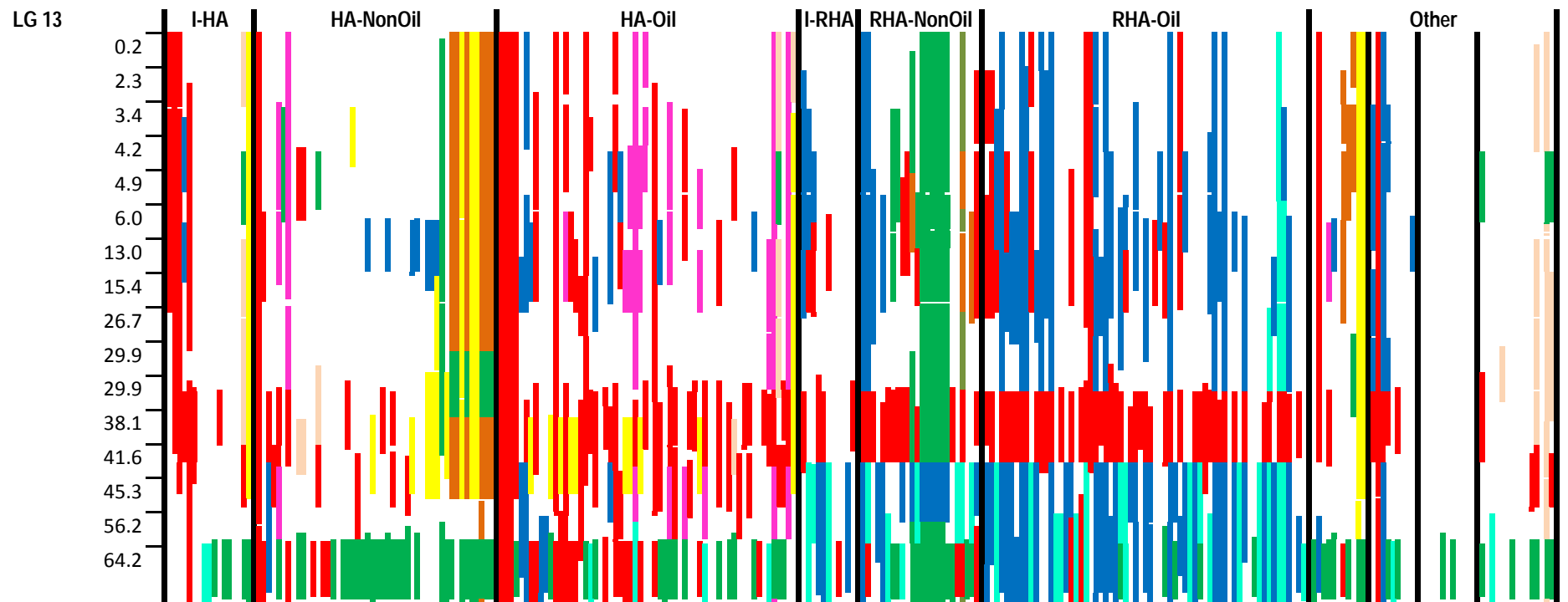

LG 14

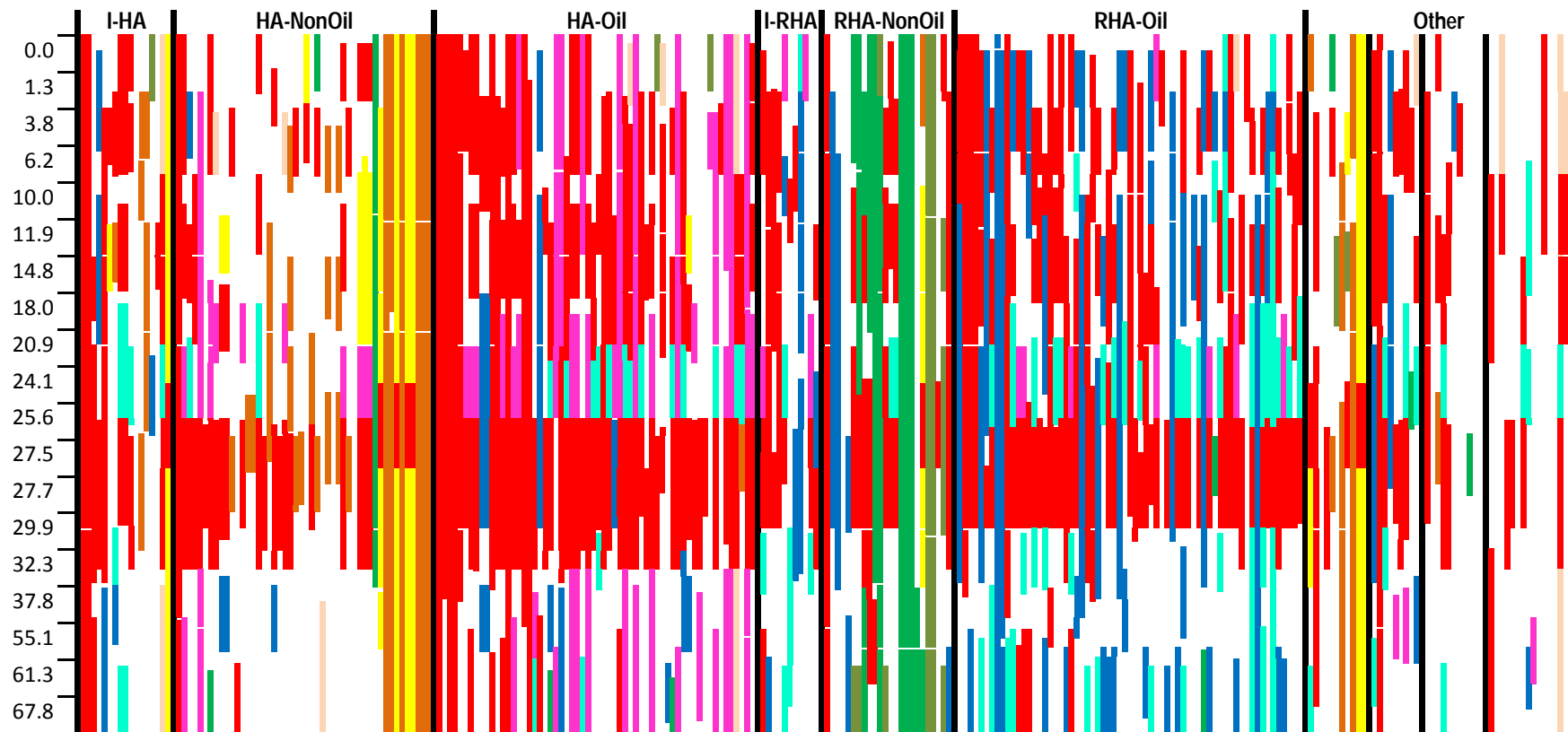

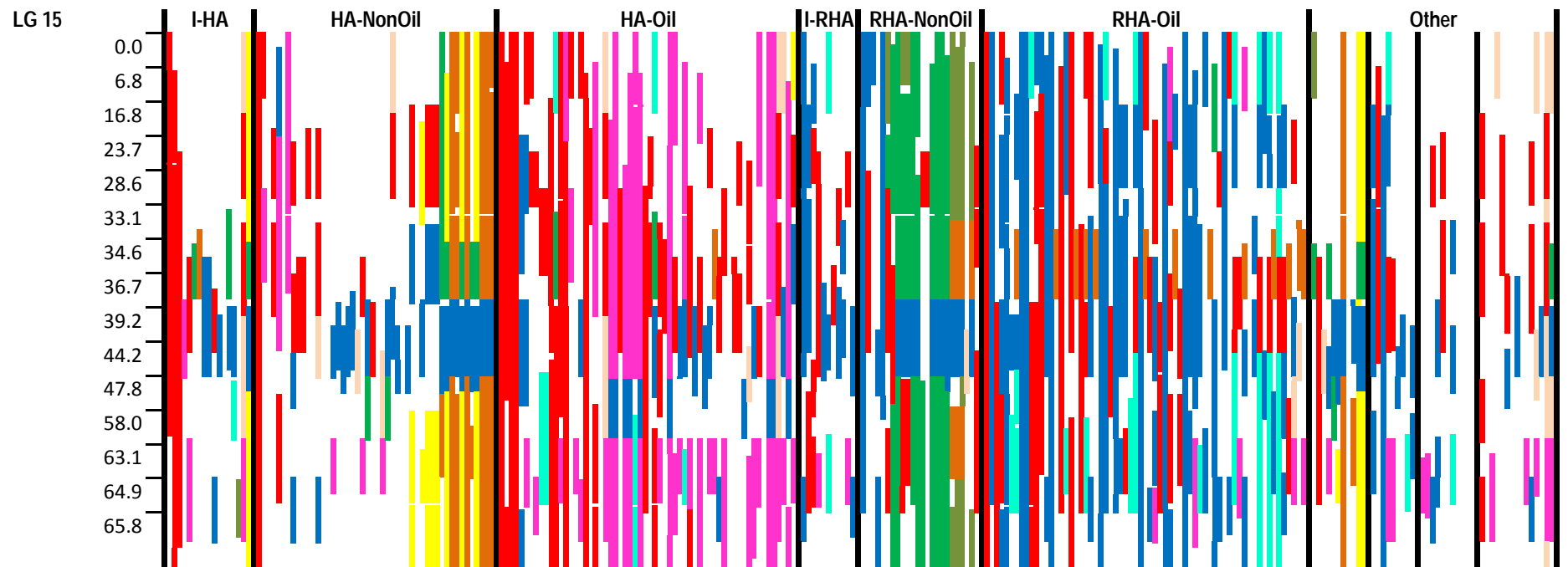

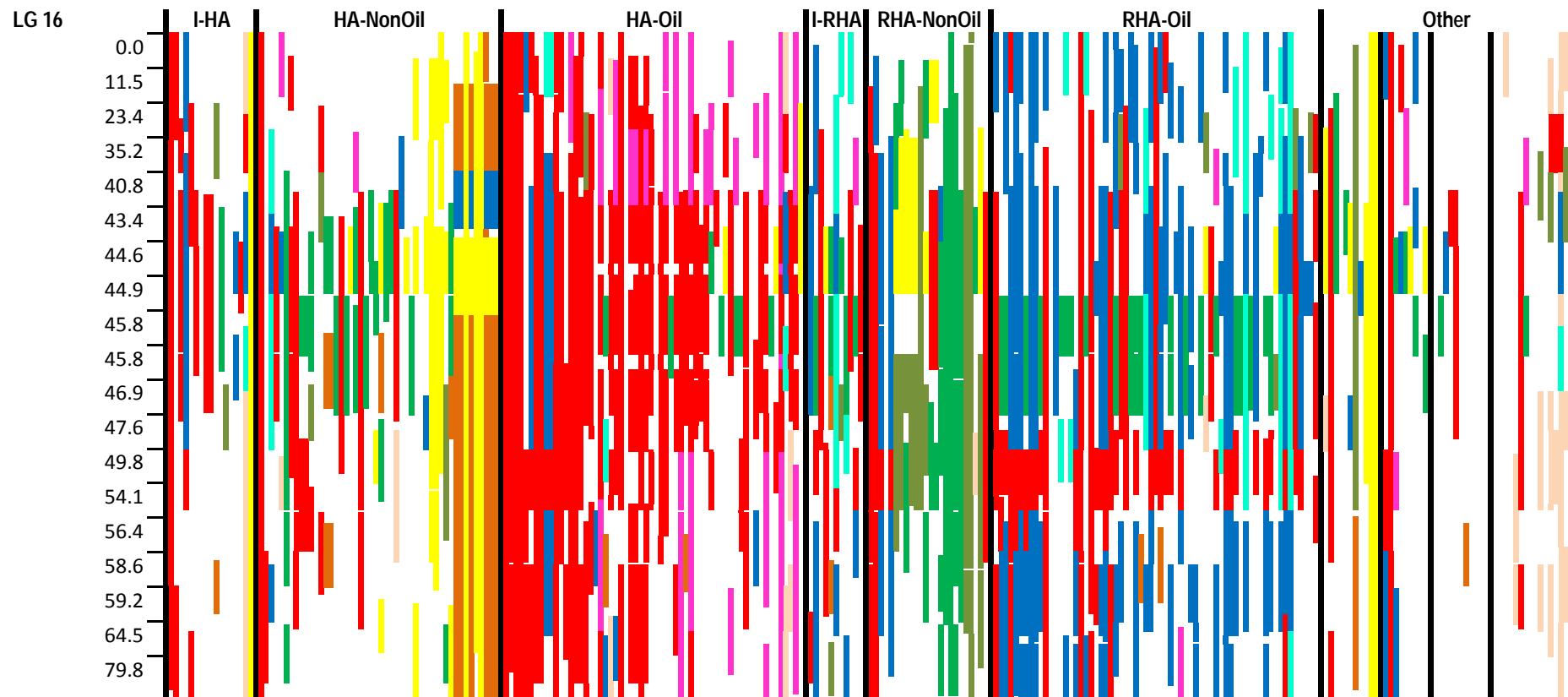

LG 17

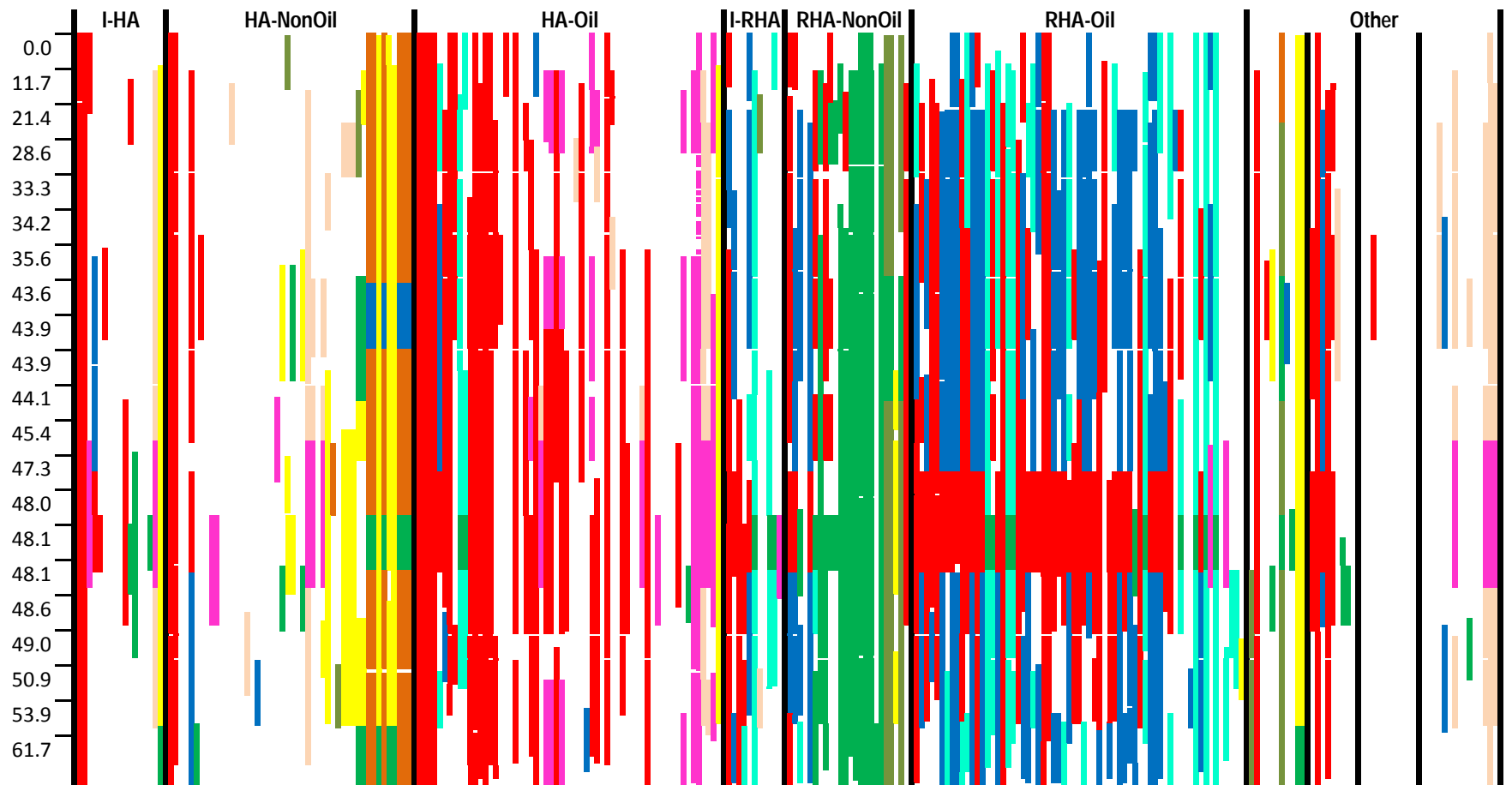

Supplement: Figure S8 — Graphical genotypes for the 17 LGs across the full population of 271 accessions. Accessions were grouped according to the previously defined accession categories as in Figure 1. The top nine genotypes were color-coded (see Table S3 for details of coding). White regions are either non-major haplotypes or regions with fewer than 25 consecutive, homozygous SNPs. (PDF) [file pgen.1003378.s008.pdf]

LG10      LG12      LG13      LG17

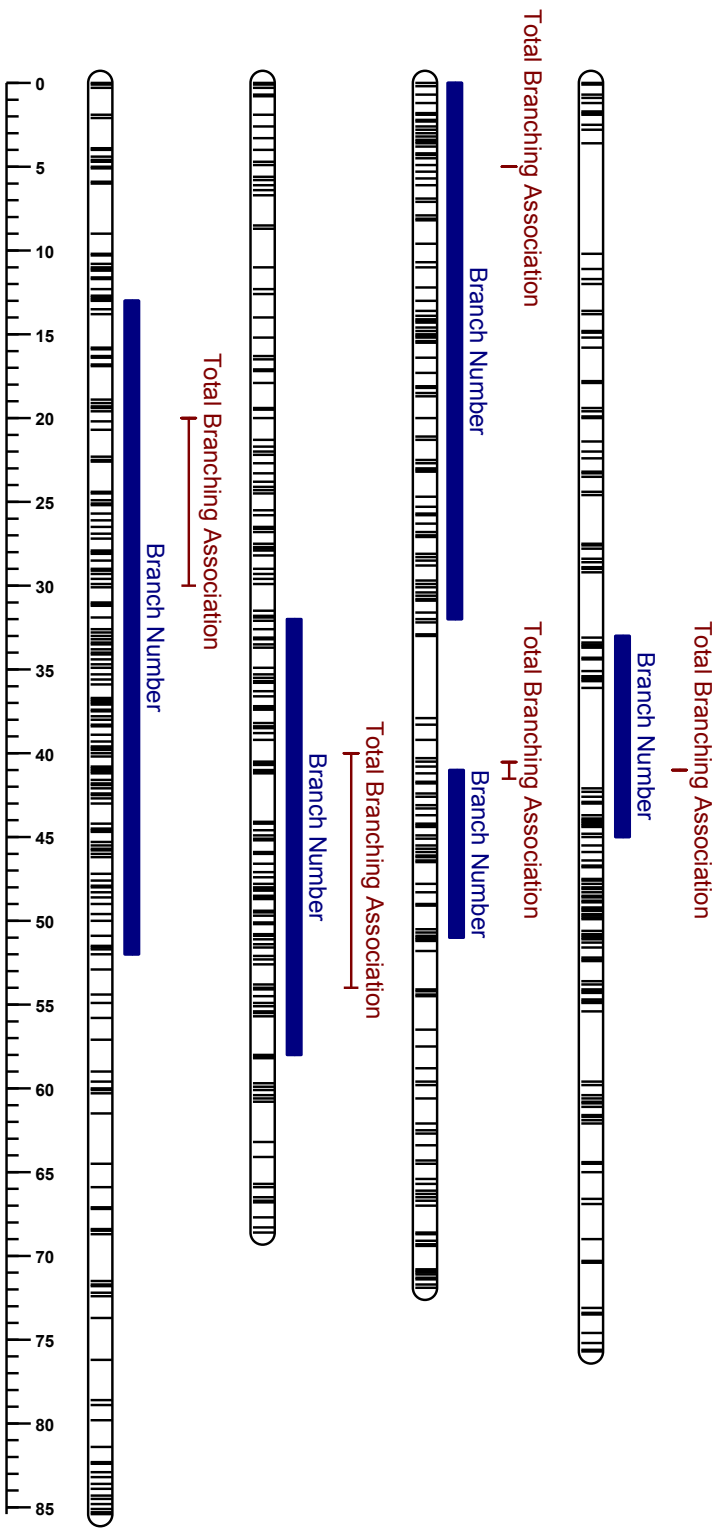

Supplement: Figure S9 — Co-localization of branching associations and QTL. Comparison of genetic map positions for significant associations and previously identified branching QTL. (PDF) [file pgen.1003378.s009.pdf]

LG9      LG17

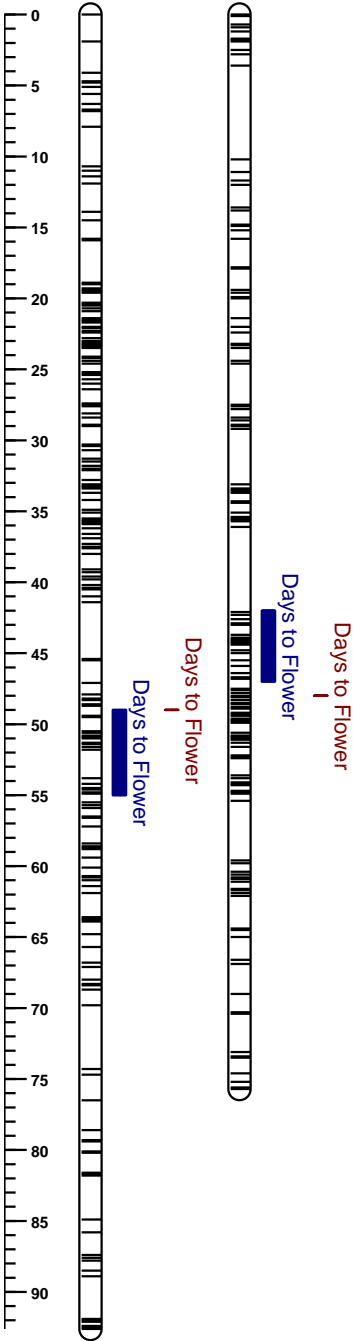

Supplement: Figure S10 — Co-localization of flowering time associations and QTL. Comparison of genetic map positions for significant associations and previously identified flowering time QTL. (PDF) [file pgen.1003378.s010.pdf]
