# Supplementary material for: Association Mapping and the Genomic Consequences of Selection in Sunflower
Source: PLoS Genet. 2013 Mar 21;9(3):e1003378. doi: 10.1371/journal.pgen.1003378 (PMC3605098; doi:10.1371/journal.pgen.1003378)
Supplement: Figure S5 — Linkage group plots of the squared allele frequency correlations (r2). Plots of the squared allele frequency correlations (r2) as a function of genetic map distance between 10,000 randomly selected pairs of SNPs separated by 50 cM or less. The red line on each graph summarizes the observed r 2 values as a function of map distance using the ksmooth function in the statistical programming language R (see text for details). (PDF) [file pgen.1003378.s005.pdf]

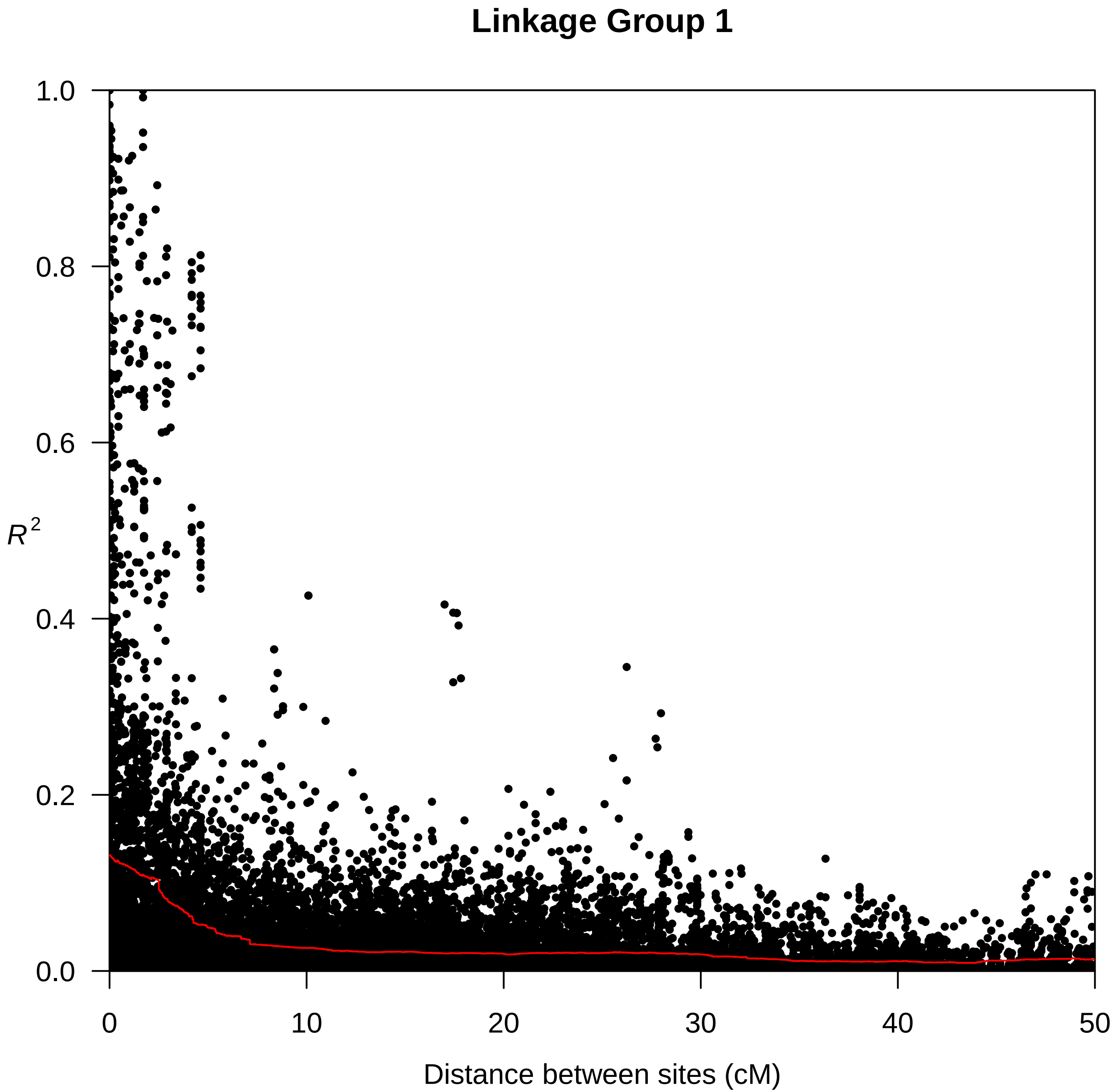
Linkage Group 1
1.0
0.8
0.6
0.4
0.2
0.0
R^2
0
10
20
30
40
50
Distance between sites (cM)

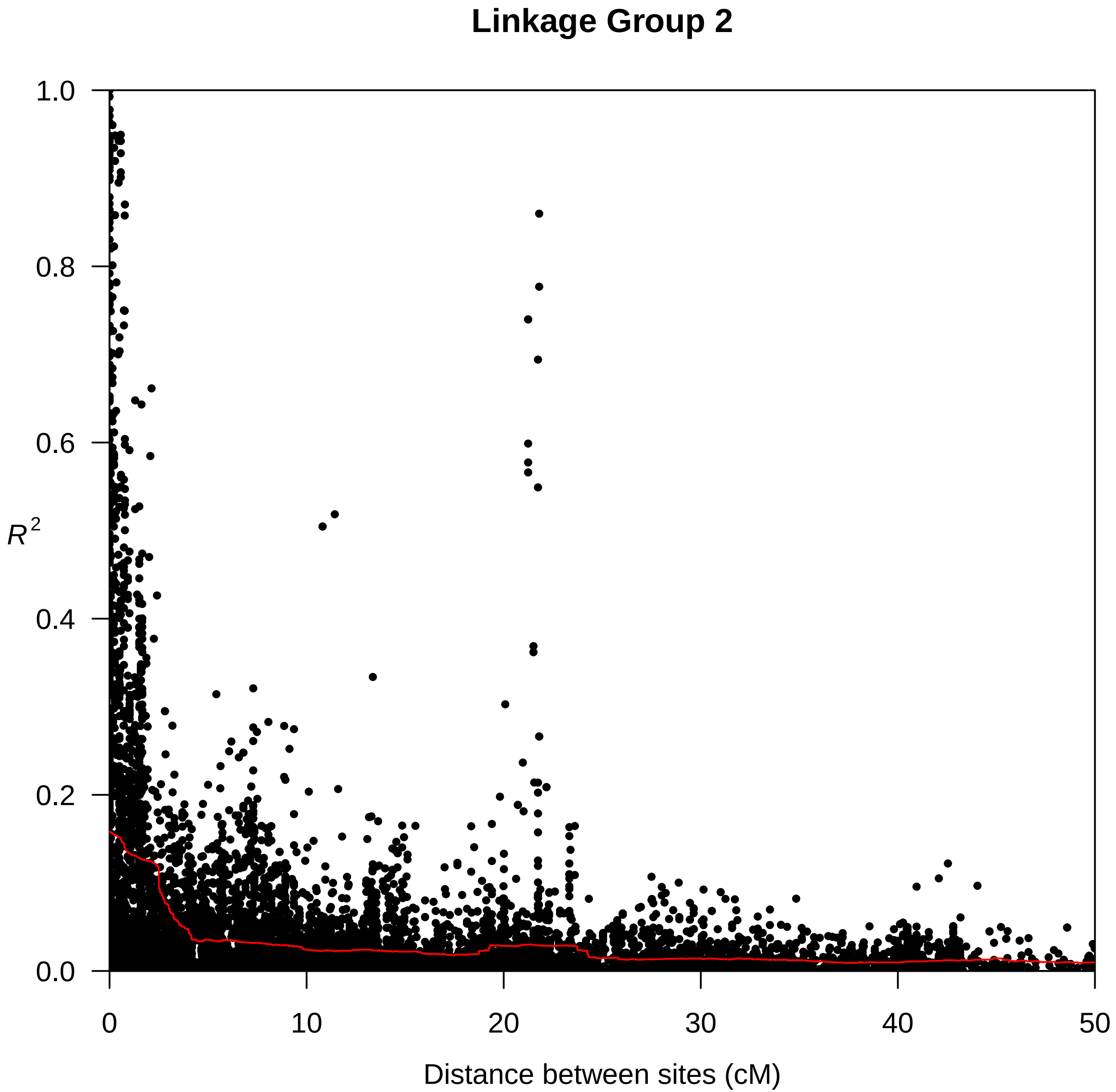

Linkage Group 2
1.0
0.8
0.6
0.4
0.2
0.0
R^2
0
10
20
30
40
50
Distance between sites (cM)

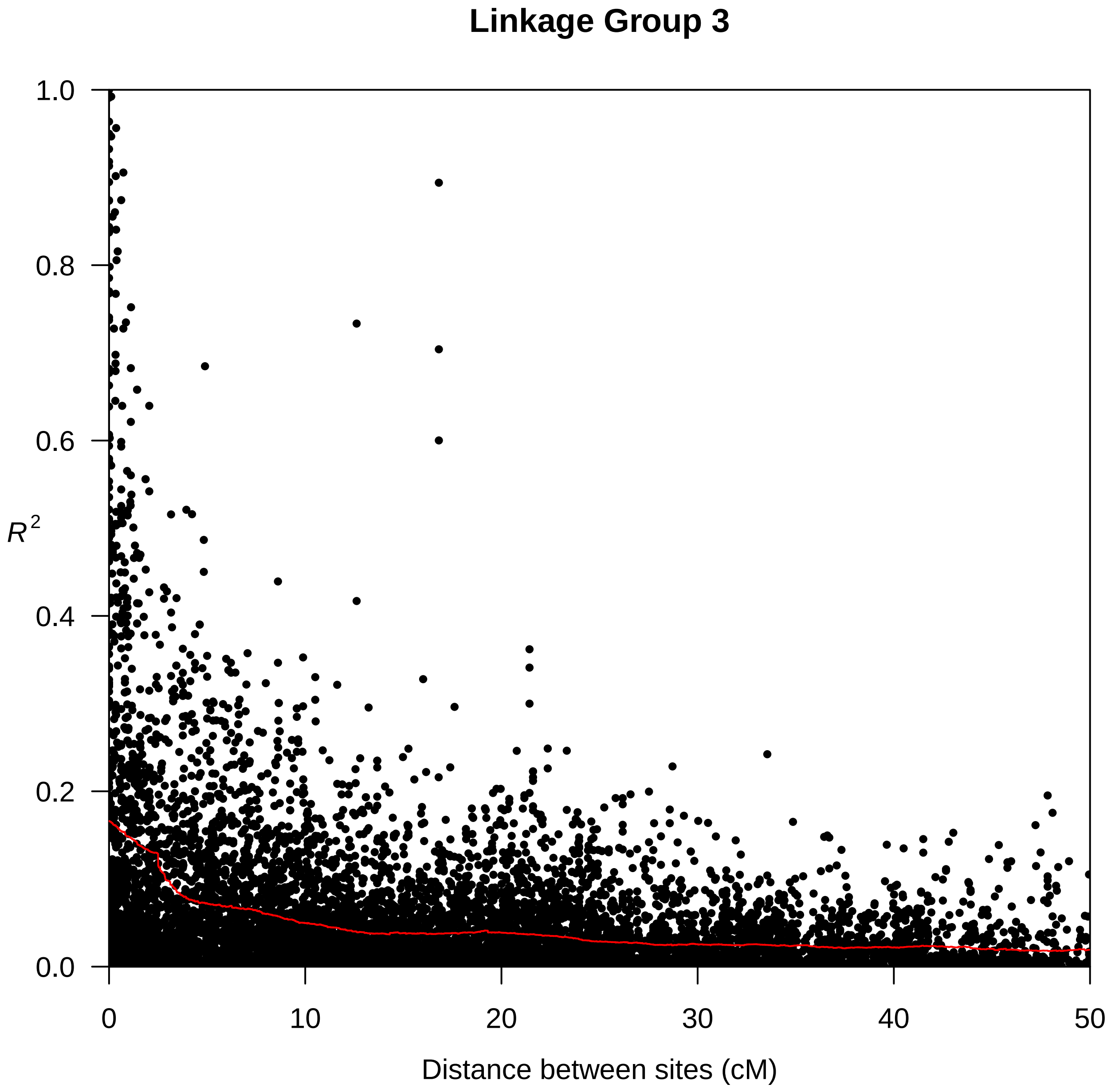
Linkage Group 3
1.0
0.8
0.6
0.4
0.2
0.0
R2
0
10
20
30
40
50
Distance between sites (cM)

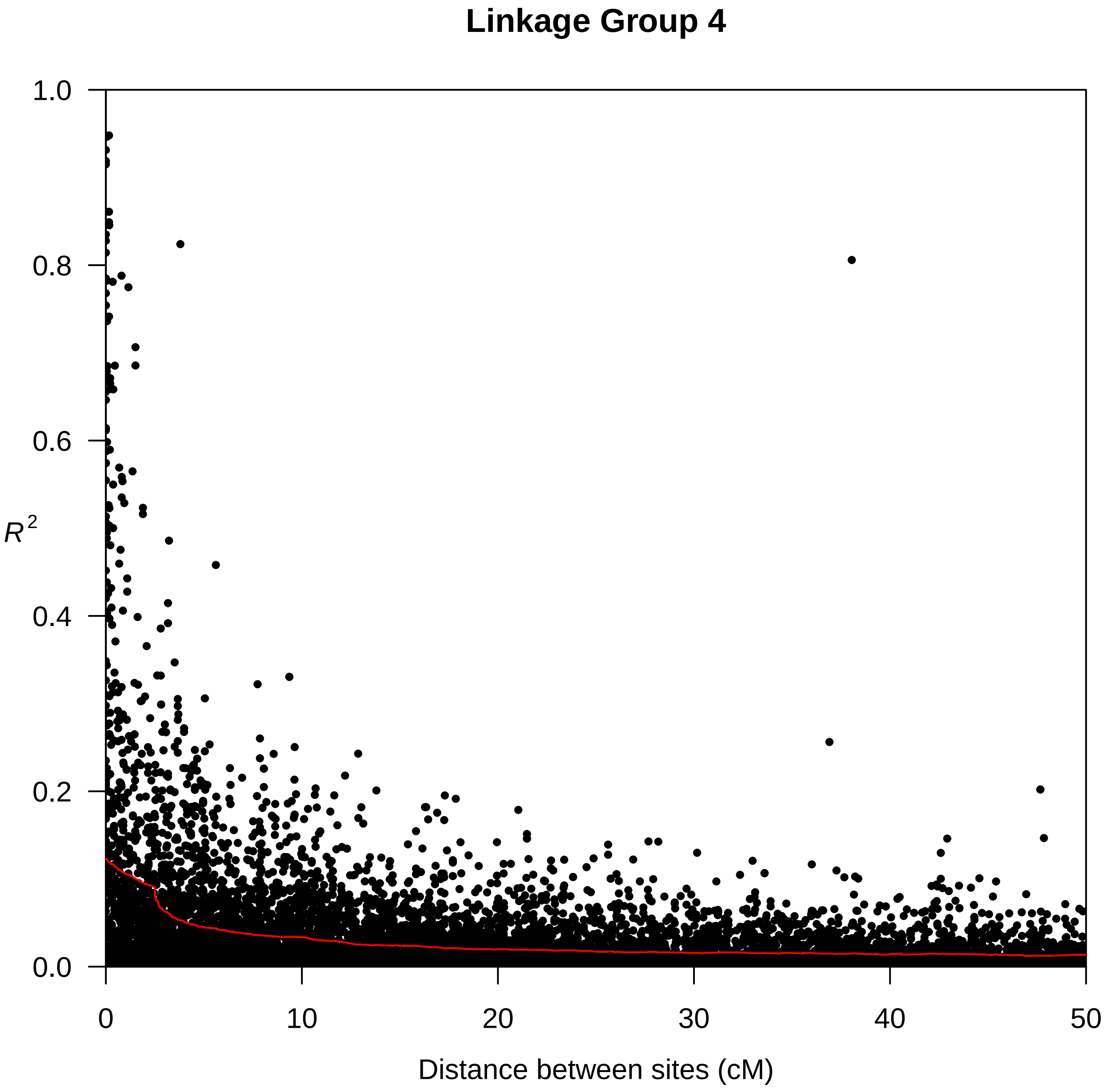

Linkage Group 4
1.0
0.8
0.6
0.4
0.2
0.0
R^2
0
10
20
30
40
50
Distance between sites (cM)

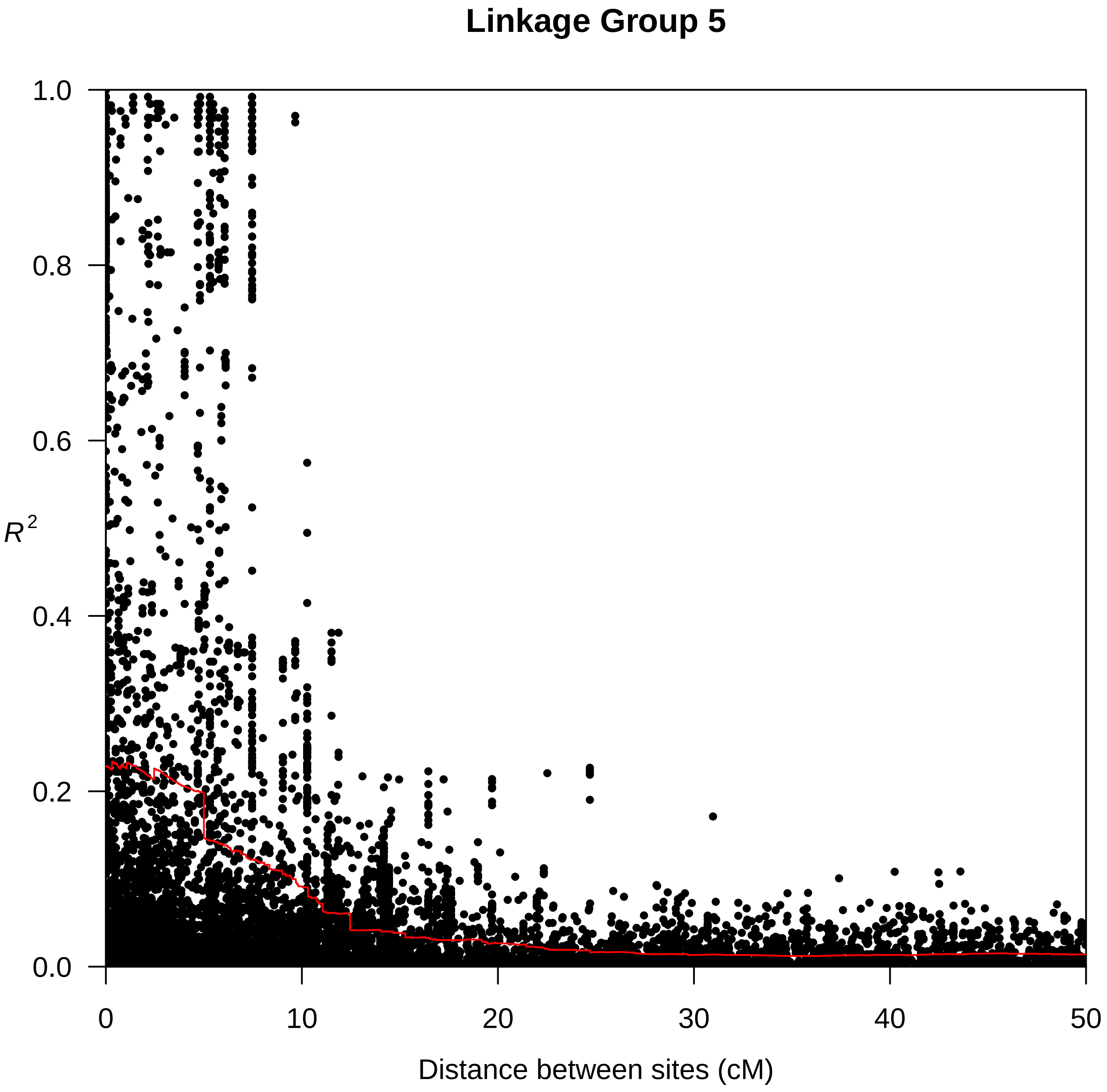
Linkage Group 5
1.0
0.8
0.6
0.4
0.2
0.0
R^2
0
10
20
30
40
50
Distance between sites (cM)

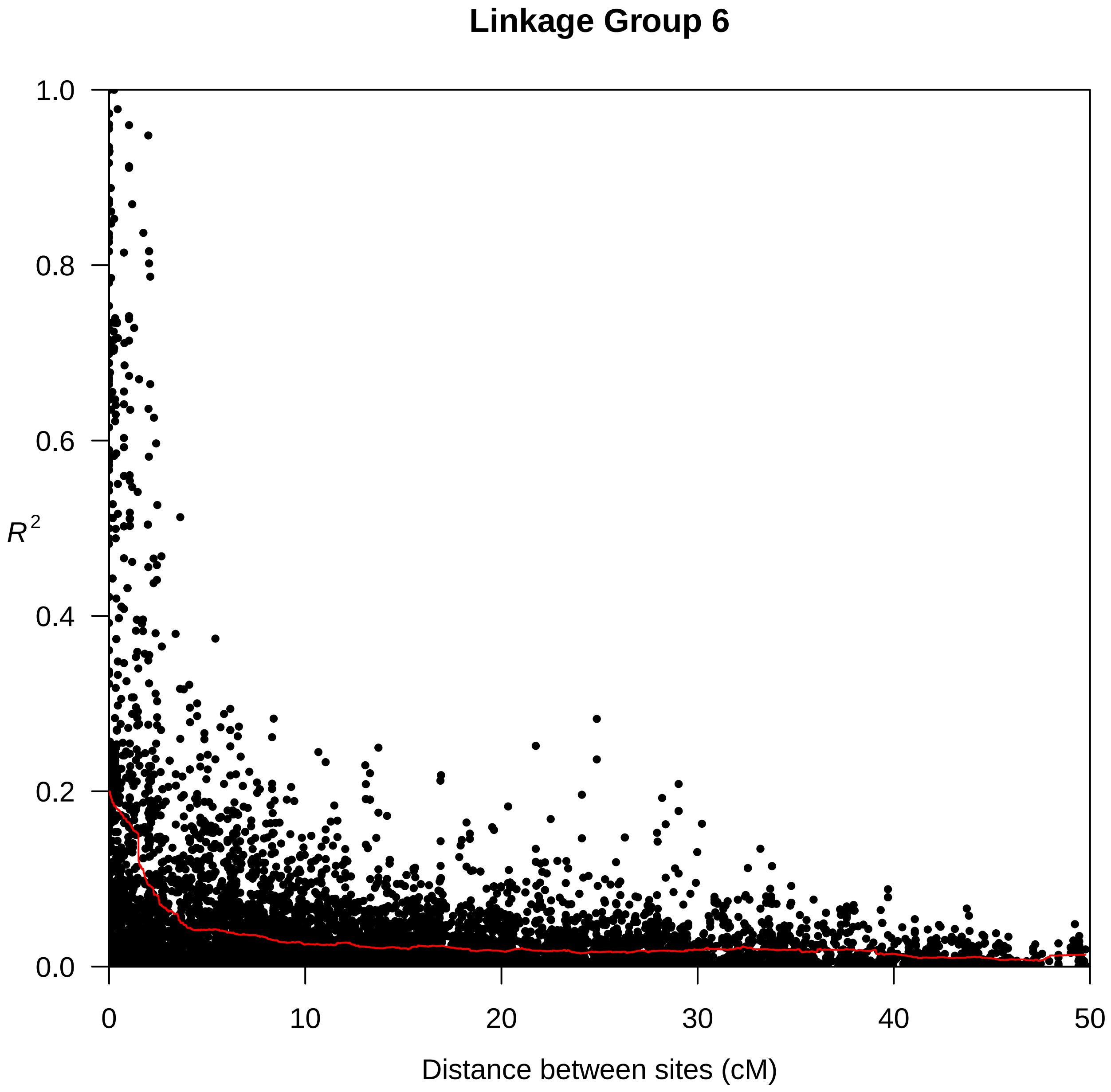

Linkage Group 6
1.0
0.8
0.6
0.4
0.2
0.0
R^2
0
10
20
30
40
50
Distance between sites (cM)

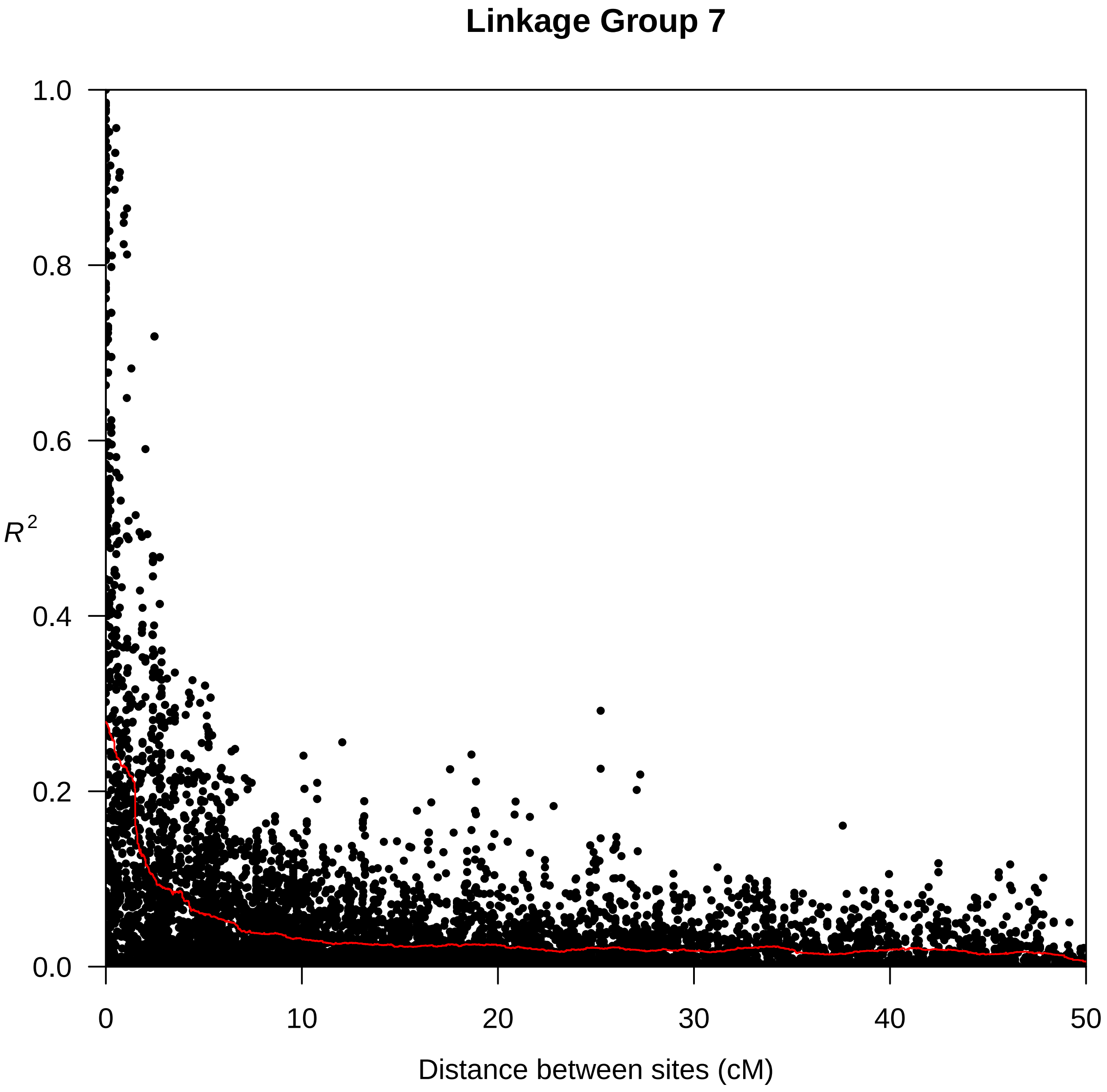

Linkage Group 7
1.0
0.8
0.6
0.4
0.2
0.0
R2
0
10
20
30
40
50
Distance between sites (cM)

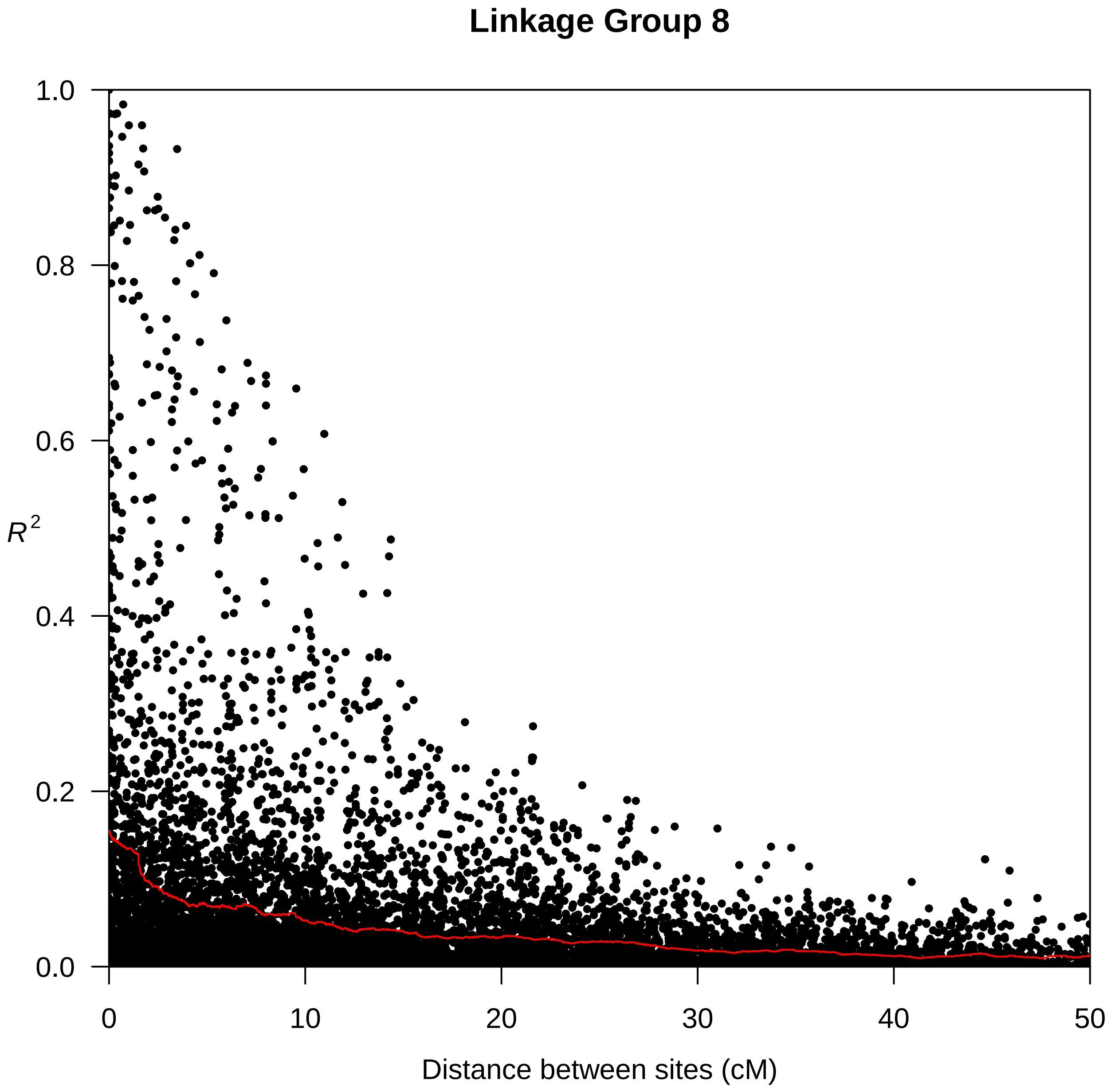

Linkage Group 8
1.0
0.8
0.6
0.4
0.2
0.0
R^2
0
10
20
30
40
50
Distance between sites (cM)

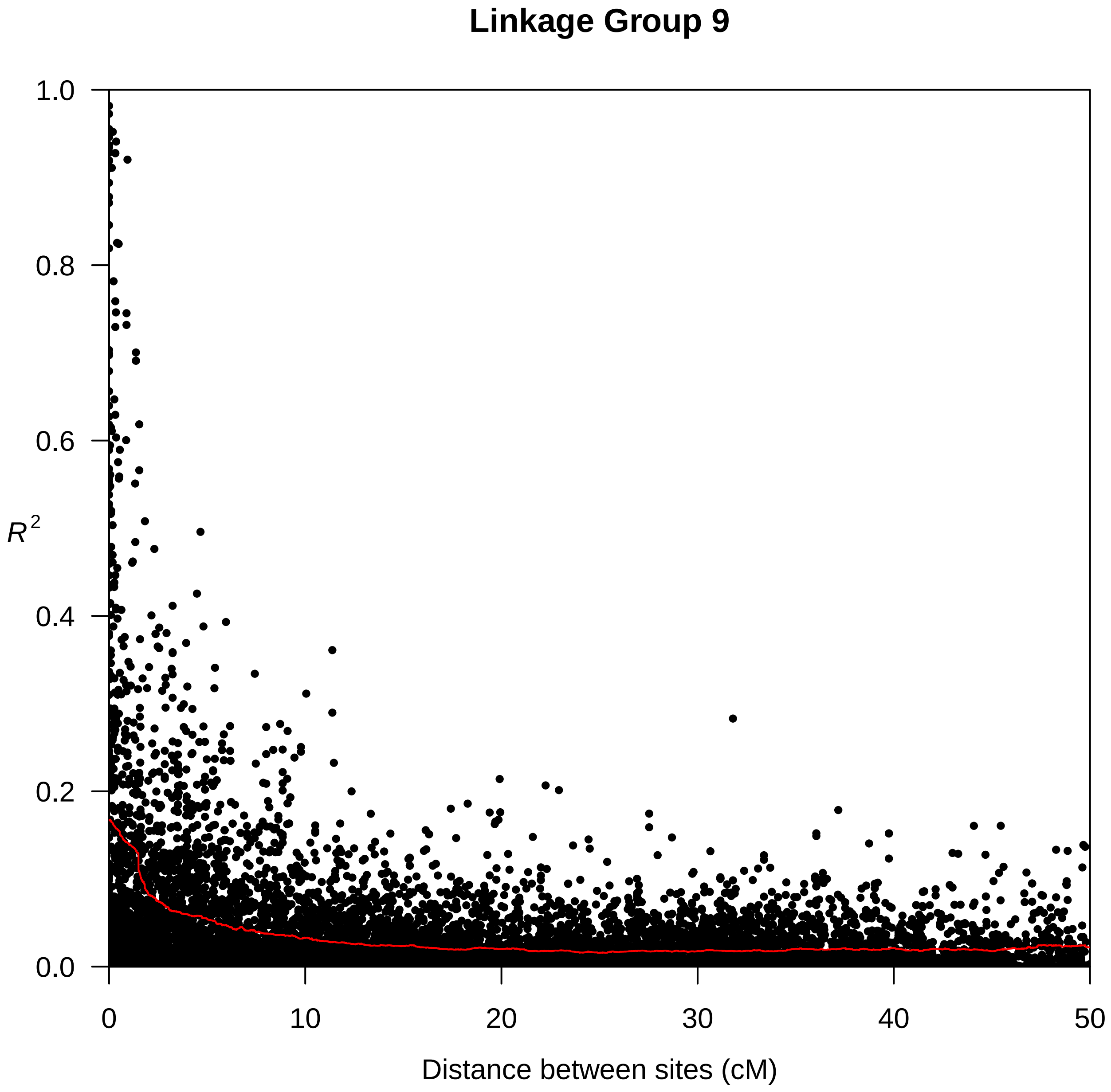

Linkage Group 9
1.0
0.8
0.6
0.4
0.2
0.0
R²
0
10
20
30
40
50
Distance between sites (cM)

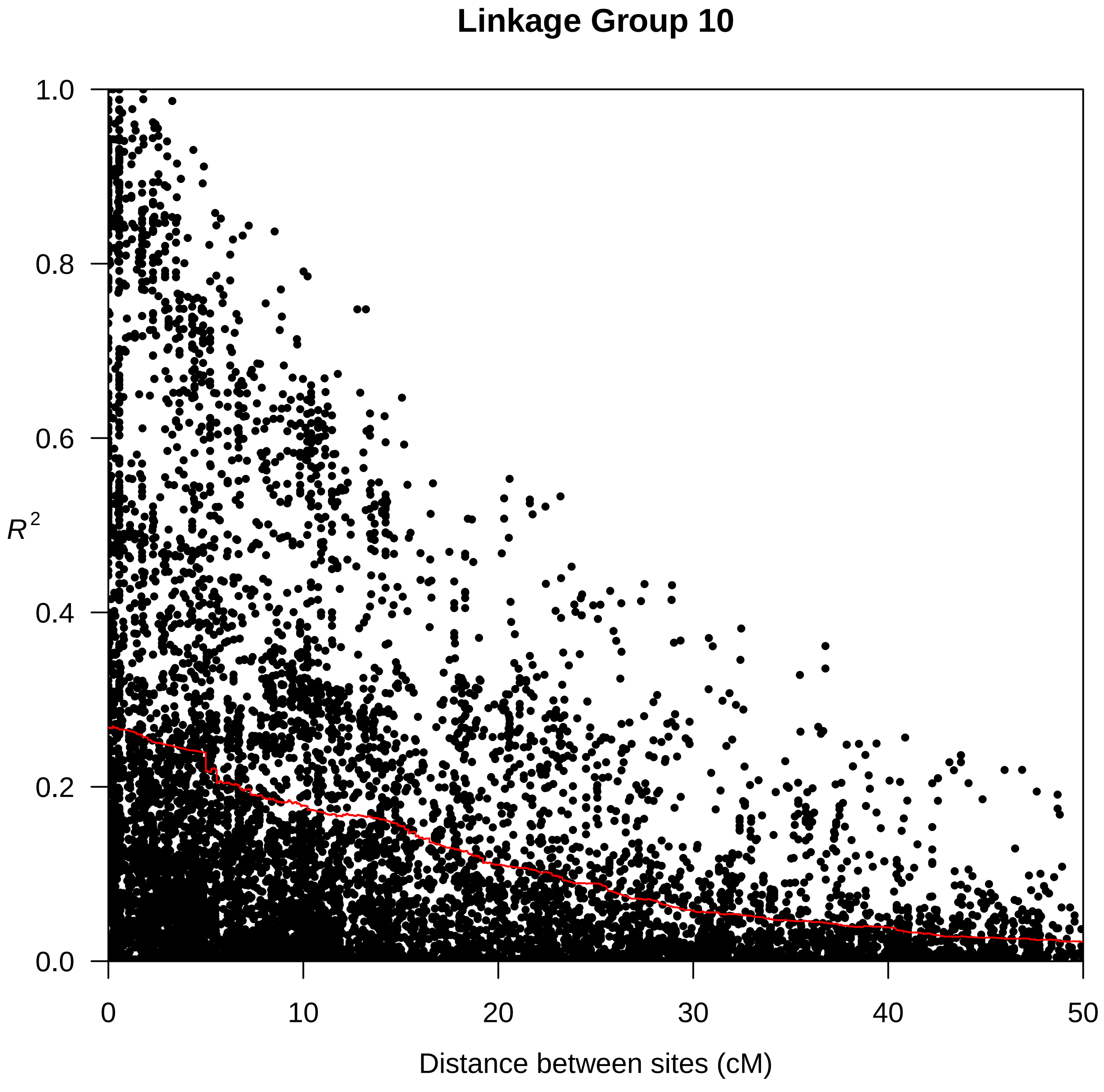
Linkage Group 10
R^2
1.0
0.8
0.6
0.4
0.2
0.0
0
10
20
30
40
50
Distance between sites (cM)

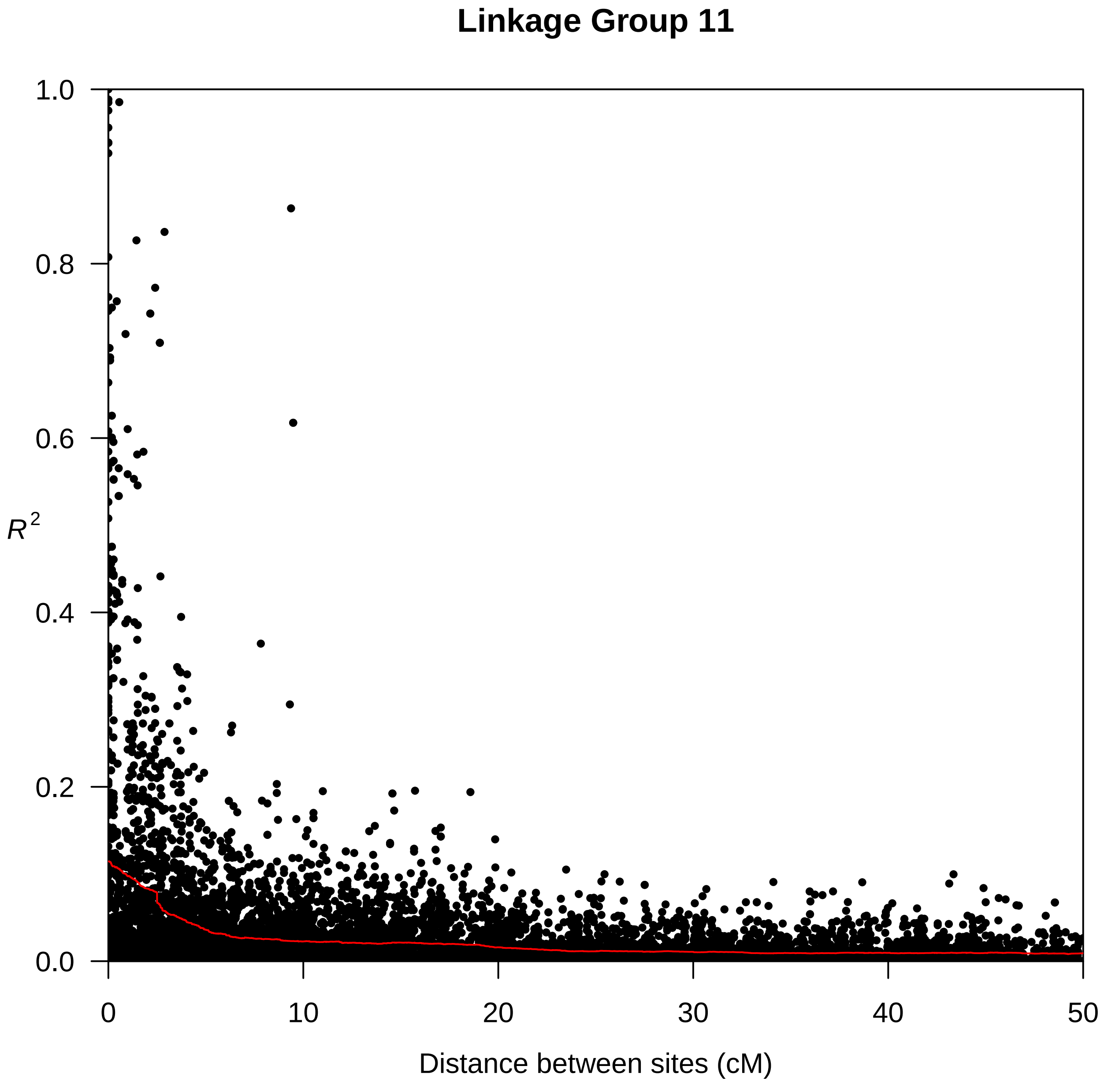

Linkage Group 11
1.0
0.8
0.6
0.4
0.2
0.0
R 2
0
10
20
30
40
50
Distance between sites (cM)

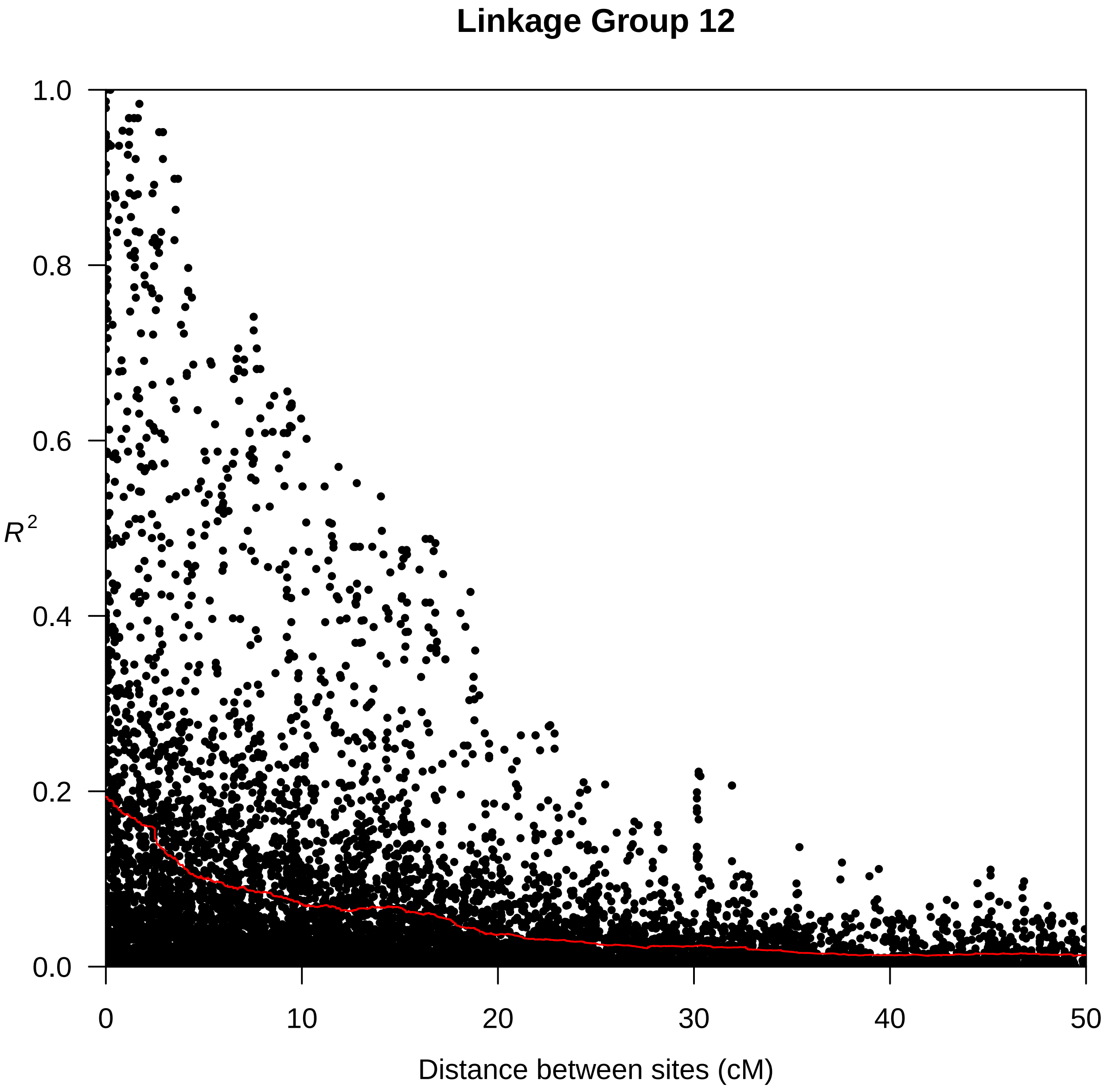

Linkage Group 12
1.0
0.8
0.6
0.4
0.2
0.0
R^2
0
10
20
30
40
50
Distance between sites (cM)

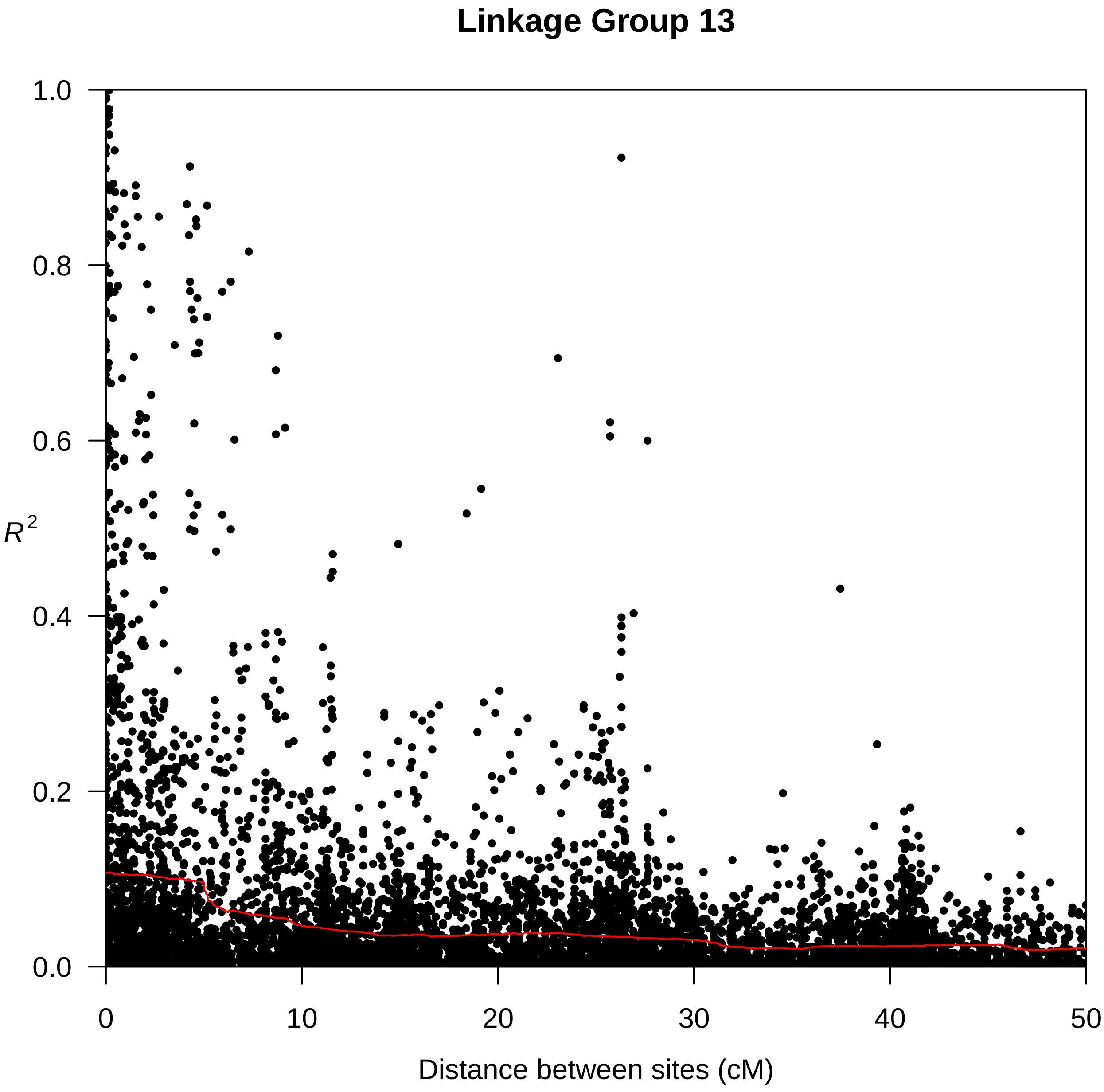
Linkage Group 13
R^2
1.0
0.8
0.6
0.4
0.2
0.0
0
10
20
30
40
50
Distance between sites (cM)

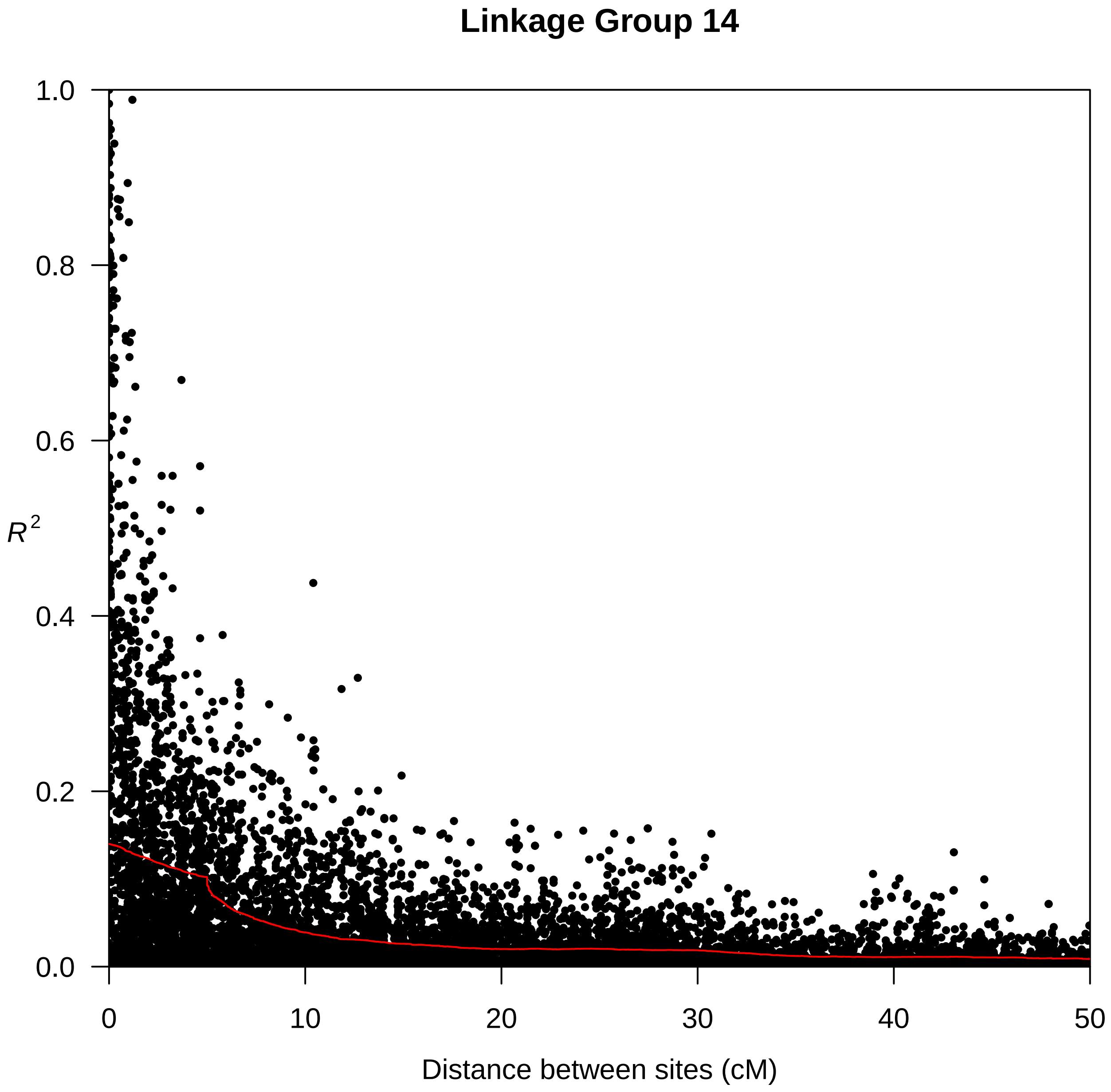
Linkage Group 14
1.0
0.8
0.6
0.4
0.2
0.0
R^2
0
10
20
30
40
50
Distance between sites (cM)

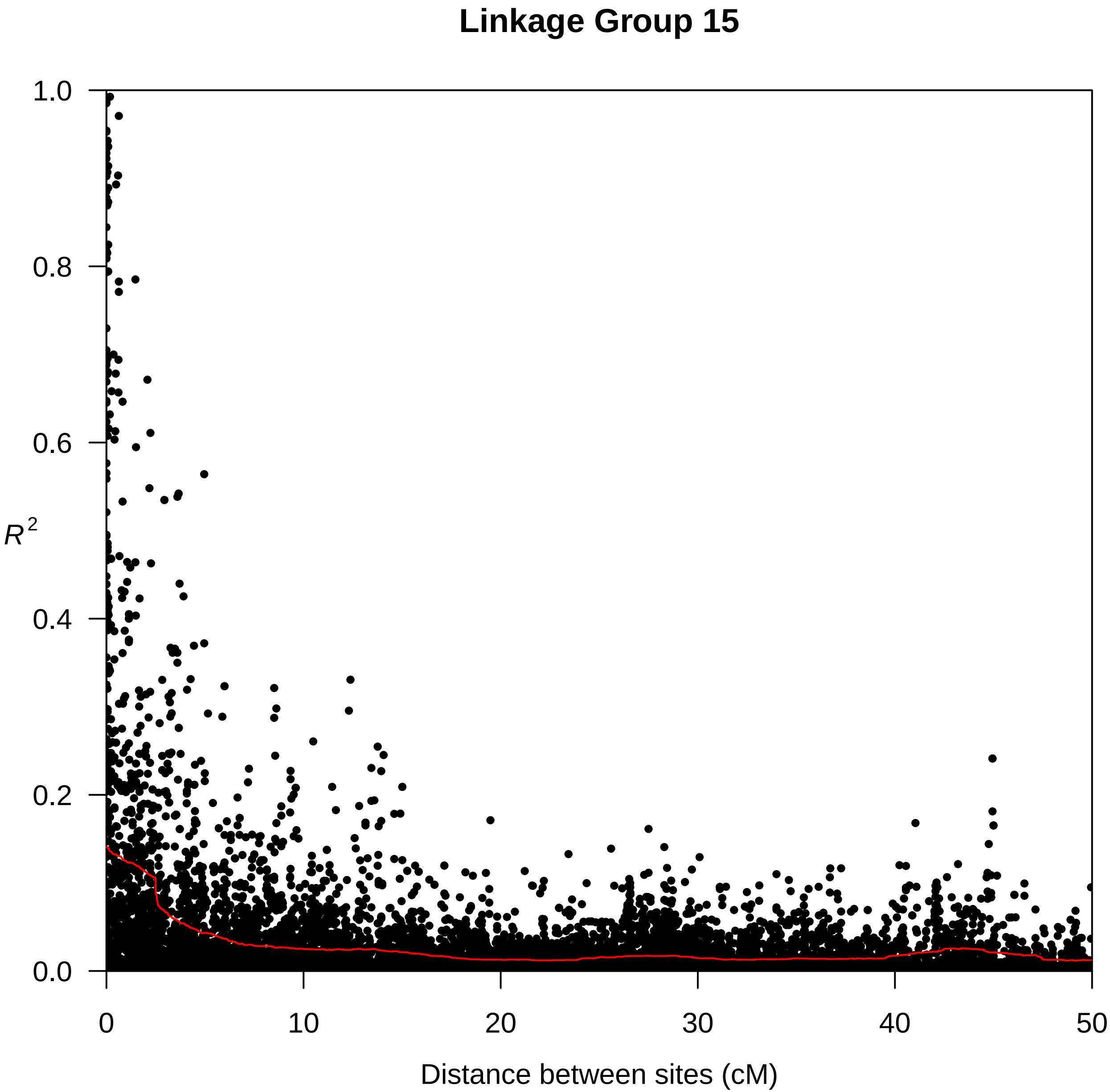
Linkage Group 15
R^2
1.0
0.8
0.6
0.4
0.2
0.0
0
10
20
30
40
50
Distance between sites (cM)

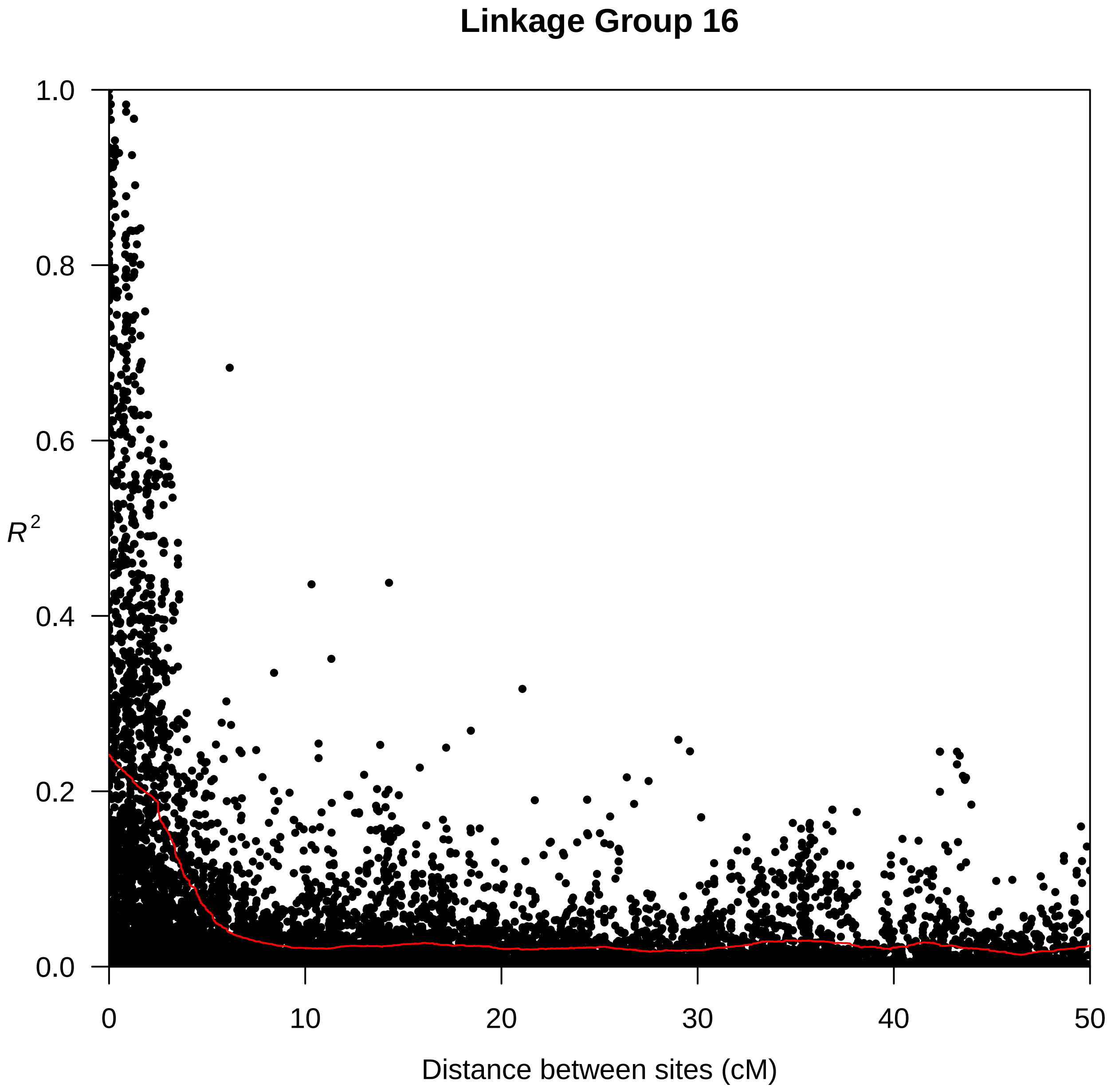
Linkage Group 16
1.0
0.8
0.6
0.4
0.2
0.0
R^2
0
10
20
30
40
50
Distance between sites (cM)

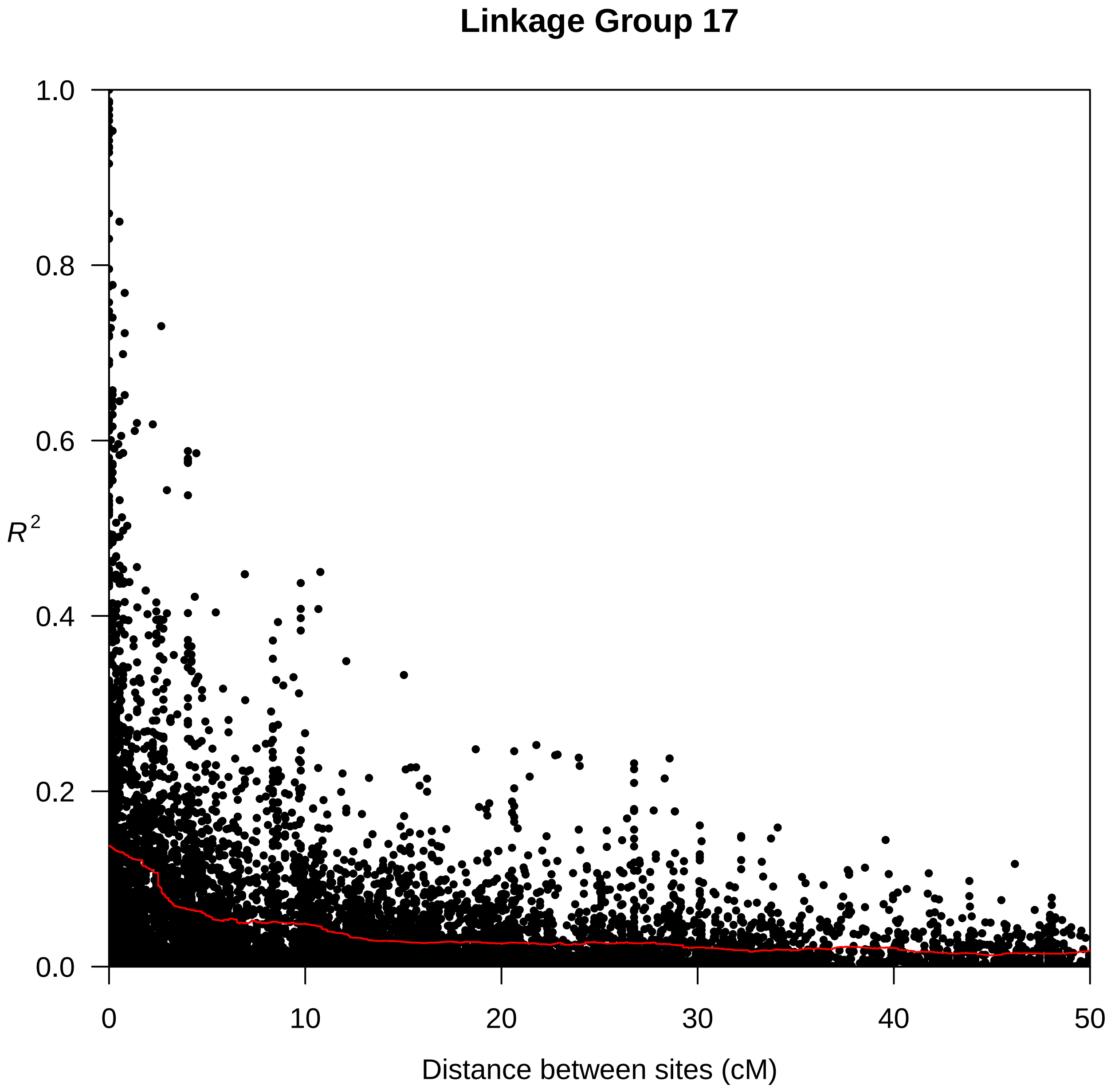
Linkage Group 17
1.0
0.8
0.6
0.4
0.2
0.0
R^2
0
10
20
30
40
50
Distance between sites (cM)
