# Supplementary material for: Association Mapping and the Genomic Consequences of Selection in Sunflower
Source: PLoS Genet. 2013 Mar 21;9(3):e1003378. doi: 10.1371/journal.pgen.1003378 (PMC3605098; doi:10.1371/journal.pgen.1003378)
Supplement: Figure S7 — Correlation matrix for the branching and flowering time (DTF) data across the three locations. The cells are color coded to indicate positive (red) or negative (blue) correlations. Significant correlations after correcting for multiple tests are starred (see text for details). (PDF) [file pgen.1003378.s007.pdf]

	GA DTF	IA DTF	BC DTF	GA Br	IA Br	BC Br
**GA DTF**	-					
**IA DTF**	0.66*	-				
**BC DTF**	0.58*	0.63*	-			
**GA Br**	-0.19*	-0.13	-0.030	-		
**IA Br**	-0.28*	-0.19*	-0.077	0.90*	-	
**BC Br**	-0.23*	-0.17*	-0.067	0.94*	0.91*	-
